# Nucleic Acid Amplification Tests for *Candida* Species Identification: A Systematic Review of Diagnostic Performance

**DOI:** 10.3390/pathogens15070753

**Published:** 2026-07-17

**Authors:** Karolina M. Czajka, Asma Bilgasem, Yamamah A. Al-Jumaili, Denver Kitching, Graham Buchan, Anu Nair, Michael Reich, Chibike Ijomah, Gopi E. Saikrishna, Chris Verschoor, Stacey A. Santi, Danielle Brabant-Kirwan, Ravi Singh, Vasu Appanna, Deborah Saunders, Sujeenthar Tharmalingam

**Affiliations:** 1 Medical Sciences Division, NOSM University, 935 Ramsey Lake Rd., Sudbury, ON P3E 2C6, Canada; kczajka@nosm.ca (K.M.C.); asma.bilgasem@mail.utoronto.ca (A.B.); yaljumaili@laurentian.ca (Y.A.A.-J.); dkitching@nosm.ca (D.K.); gbuchan@nosm.ca (G.B.); anair3@laurentian.ca (A.N.); mreich2@laurentian.ca (M.R.); cijomah@laurentian.ca (C.I.); gsaikrishna@nosm.ca (G.E.S.); cverschoor@hsnri.ca (C.V.); ssanti@hsnri.ca (S.A.S.); dbrabantkirwan@hsnsudbury.ca (D.B.-K.); rsingh@hsnsudbury.ca (R.S.); dsaunders@hsnsudbury.ca (D.S.); 2 School of Natural Sciences, Laurentian University, Sudbury, ON P3E 2C6, Canada; vappanna@laurentian.ca; 3 Health Sciences North Research Institute, Sudbury, ON P3E 2H2, Canada

**Keywords:** *Candida albicans*, *Candidozyma auris*, molecular diagnostics, nucleic acid amplification tests, PCR, isothermal amplification, antifungal resistance, species identification, point-of-care diagnostics, diagnostic performance, systematic review

## Abstract

Rapid and accurate identification of *Candida* species is critical for guiding antifungal therapy, especially with the emergence of intrinsically resistant pathogens. However, diagnostics using culture-based methods remain slow and labor-intensive, limiting timely treatment decisions. This systematic review evaluated the diagnostic performance and clinical applicability of nucleic acid amplification tests (NAATs) for *Candida* species identification using a PubMed search completed on 23 June 2025. A total of 888 records were screened, 333 full-text articles were assessed, and 158 studies were included based on criteria including comparison with standard diagnostic methods, diagnostic performance reporting, and involvement of clinical samples. PCR-based approaches were the most widely used, including conventional, nested, multiplex, real-time, and droplet digital PCR. Isothermal methods such as loop-mediated isothermal amplification (LAMP) and recombinase polymerase amplification (RPA) were increasingly represented. Common molecular targets included the ITS and 18S/28S rDNA regions. The risk of bias assessment was completed with the QUADAS-2 tool. Diagnostic performance varied depending on assay design, specimen type, gene target, and reference method. Excellent specificity and low limits of detection were achieved, especially with isothermal platforms offering the shortest turnaround times and greatest potential for point-of-care implementation. Multiplex assays were particularly advantageous for detecting mixed-species samples, while highly specific assays were optimal for distinguishing clinically important species such as *Candidozyma auris*, *Nakaseomyces glabratus*, and *Pichia kudriavzevii*. Overall, NAATs represent a promising diagnostic tool for *Candida* species identification, but broader clinical adoption will require improved standardization, validation across diverse patient populations, and clearer interpretation of fungal burden in the context of colonization versus infection.

## 1. Introduction

### 1.1. Background on Candida Infection

*Candida* infections are caused by yeasts that commonly colonize human mucosal surfaces but can become opportunistic pathogens when host immunity or normal microbiota are out of balance [1]. They are of significant public health concern, particularly among immunocompromised populations such as individuals living with HIV, cancer patients undergoing chemotherapy, and organ transplant recipients receiving immunosuppressive therapy [1,2]. If acute infections are not treated appropriately in good time, the fungal infection can invade the host’s bloodstream (candidemia) or body tissues. Invasive candidiasis accounts for a substantial proportion of healthcare-associated bloodstream infections globally, increasing morbidity, length of hospital stay, and healthcare costs [1,3].

Globally, *Candida albicans* is considered the most common cause of the fungal infection candidiasis [4]. For example, *C. albicans* accounts for 40–70% of candidemia cases depending on the geographic region [5]. Typically, a commensal organism in a healthy host, this opportunistic pathogen can transform into a pathogenic state with hyphal phenotypes and biofilm formation when the host immune response is compromised [6]. While *C. albicans* strains are usually susceptible to azole antifungals, increasing rates of resistance have been cited, particularly in isolates from patients with chronic infections receiving long-term antifungal therapy [7]. Resistance rates can also vary by geographic region and are usually estimated at 5–10% [8,9].

In contrast, non-albicans *Candida* (NAC) species are phylogenetically diverse, and numerous species are intrinsically resistant to first-line antifungal treatments [10]. *Nakaseomyces glabratus* (recently renamed from *Candida glabrata* for improved taxonomic classification) and *Pichia kudriavzevii* (formerly *Candida krusei*) are more closely related to *Saccharomyces cerevisiae* than *C. albicans* and are both intrinsically resistant to fluconazole (FLZ) [11,12]. Of particular concern is *Candidozyma auris* (*Candida auris*), a globally emerging species with high transmissibility in healthcare environments. It is multidrug-resistant with high resistance to fluconazole and increasing resistance to amphotericin B and echinocandins [13]. Other clinically relevant NAC such as *Candida tropicalis* and *Candida parapsilosis* are in the CTG clade with *C. albicans.* Increasing rates of acquired resistance have been reported for these species up to 50% and 10%, respectively [14,15].

Rapid and accurate species-level identification is needed to address the challenges in diagnosis and treatment like indistinguishable clinical phenotypes between *Candida* species and increasing antifungal resistance rates. Timely identification of species informs appropriate antifungal selection, which can increase treatment success and ultimately improve patient outcomes.

### 1.2. Reference Methods of Candida Species Identification

Currently, culture-based methods are part of the usual reference standard for diagnosing *Candida* infections. However, they are limited by long turnaround times, moderate sensitivity (~50%, range 21–71%), and occasional false positives (5–10%) due to contamination or colonization [16,17,18].

Diagnostic NAAT performance is typically evaluated against a reference method, most commonly sample culturing followed by MALDI-TOF mass spectrometry analysis [19,20,21,22]. Widely used systems such as Vitek 2 (bioMérieux, Marcy-l’Étoile, France) and Vitek MS (bioMérieux, Marcy-l’Étoile, France) offer high accuracy but are time-consuming and costly to maintain [23]. Other reference methods include CHROMagar *Candida* (CHROMagar, Neogen Genomics, Paris, France) and BD Affirm (Becton Dickinson, Franklin Lakes, NJ, USA) [24,25,26]. Alternatively, some studies used multiplex PCR or sequencing-based NAAT methods to evaluate other species identification assays [26]. An important consideration is that the feasibility of a reference method can vary across laboratories depending on resource constraints and other limiting factors.

### 1.3. Rationale and Objectives for the Review

Traditional culture-based methods, despite being the historical gold standard, are limited by time delays and variable diagnostic performance. Over the past twenty years, substantial advances in NAATs have helped to overcome these limitations, enabling rapid and accurate identification of *Candida* species. Despite the development of NAAT-based assays, their diagnostic accuracy and clinical utility have yet to be comprehensively assessed, particularly considering rising antifungal resistance.

This review aims to evaluate the current literature on NAATs used for *Candida* species identification, with an emphasis on analytical sensitivity and specificity. Practical considerations, including speed, cost, and feasibility of clinical implementation, will also be discussed. By highlighting the most promising NAAT-based techniques, this review aims to support their integration into clinical practice and enhance the accuracy of diagnosis and subsequent treatment decisions.

## 2. Materials and Methods

### 2.1. Systematic Review Eligibility Criteria

This systematic review was conducted following the PRISMA-ScR guidelines (2018). Eligible studies were peer-reviewed journal articles published in English between 1 January 2005 and 23 June 2025. No restrictions were applied based on age, sex, or geographic location.

Studies were included if they: (i) focused on the development or application of NAAT-based methods for *Candida* species identification; (ii) evaluated novel NAAT approaches or compared NAATs with standard diagnostic methods; (iii) reported at least one diagnostic performance metric, such as sensitivity or specificity; (iv) involved human subjects with confirmed or suspected *Candida* infection, or related clinical presentations such as sepsis, and included analysis of clinical samples such as isolates or positive blood culture samples; and (v) were available in English or as an English-translated version.

Studies were excluded if they: (i) did not focus on *Candida* species identification; (ii) were limited to theoretical models or simulation-based approaches without clinical validation; (iii) lacked experimental data or diagnostic performance outcomes (e.g., sensitivity, specificity, or time to result); (iv) were review articles; or (v) were case reports.

### 2.2. Systematic Search Strategy

A systematic search of the PubMed database was performed on 23 June 2025, using three keyword clusters related to *Candida* infections, diagnostics, and nucleic acid detection. Keywords were combined with logical operators (AND, OR) and applied to titles and abstracts.

PubMed search query:

(“*Candida*”[Title/Abstract]) AND (“nucleic acid amplification”[Title/Abstract] OR “PCR”[Title/Abstract] OR “polymerase chain reaction”[Title/Abstract] OR “real-time PCR”[Title/Abstract] OR “qPCR”[Title/Abstract] OR “RT-PCR”[Title/Abstract] OR “isothermal amplification”[Title/Abstract] OR “LAMP”[Title/Abstract] OR “loop-mediated amplification”[Title/Abstract] OR “RPA”[Title/Abstract] OR “recombinase polymerase amplification”[Title/Abstract] OR “NASBA”[Title/Abstract] OR “nucleic acid sequence-based amplification”[Title/Abstract] OR “TMA”[Title/Abstract] OR “transcription-mediated amplification”[Title/Abstract] OR “droplet digital PCR”[Title/Abstract] OR “ddPCR”[Title/Abstract] OR “next generation sequencing”[Title/Abstract] OR “NGS”[Title/Abstract]) AND (“diagnosis”[Title/Abstract] OR “diagnostic”[Title/Abstract] OR “sensitivity”[Title/Abstract] OR “specificity”[Title/Abstract] OR “performance”[Title/Abstract]).

**Figure 1:**
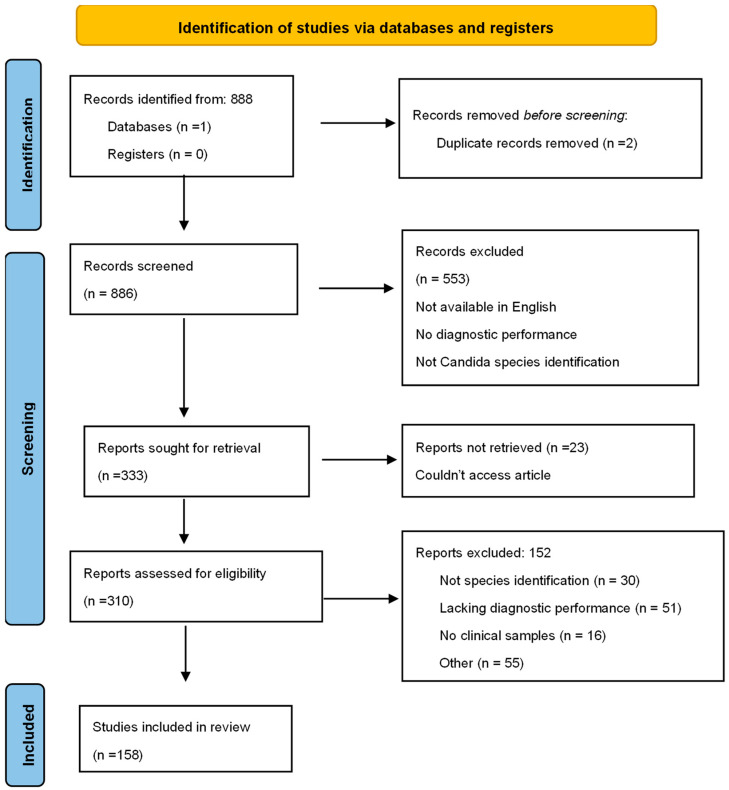
PRISMA flow diagram displaying the study selection process for the systematic review. First, records were identified through the PubMed database and screened based on their abstracts, followed by full-article assessment for final inclusion in the review based on the eligibility criteria.

Review articles were excluded using the “NOT Review” filter. The search yielded 888 records; 555 were excluded during title and abstract screening, leaving 333 articles for full-text review. Of these, 175 were excluded, resulting in 158 studies meeting the final eligibility criteria. To minimize bias, two independent reviewers screened all records at both the abstract and full-text stages. Discrepancies were resolved through discussion and, when appropriate, inclusion in subsequent review rounds. Data for included studies were extracted during full-text review using targeted keyword searches. The primary outcome was diagnostic performance of species identification assays. Additional variables included NAAT gene targets, clinical sample types, and disease context. Missing data were left unreported in the tables. The review was not registered in PROSPERO, and no review protocol was prepared. The PRISMA flow diagram displaying the search strategy results is presented in Figure 1. The PRISMA checklist for this systematic review is provided in Supplementary S1.

### 2.3. Quality Assessment of Included Studies

The risk of bias assessment was completed using the Quality Assessment of Diagnostic Accuracy Studies (QUADAS-2) tool for diagnostic performance (https://www.bristol.ac.uk/media-library/sites/quadas/migrated/documents/quadas2.pdf URL (accessed on 6 May 2026)). Two independent reviewers assessed each study for the risk of bias. The QUADAS-2 tool focuses on the following 4 domains: (A) patient selection, (B) index test, (C) reference standard, (D) flow and timing. All 4 domains considered the risk of bias, and domains 1–3 also assessed concerns regarding applicability. Each study was assigned a score for each domain, e.g., high, some concerns, or low. The summary plot and traffic plot were generated with the robvis tool [27].

## 3. Results and Discussion

A total of 158 papers were identified for inclusion in this systematic literature review of NAATs used for *Candida* species identification. The Results and Discussion sections are organized by NAAT type. Within each section, subsections first describe (i) the principles of the nucleic acid amplification test and the corresponding amplicon generation/visualization, followed by (ii) a synthesis of the relevant studies with diagnostic performance and a discussion of key findings.

A wide range of sample sizes were observed across the included studies, reflecting substantial variability in study scale. The most frequently reported NAAT platforms were PCR-based methods, encompassing conventional PCR (17 articles), LATE-PCR (1 article), nested PCR (9 articles), dye-based qPCR (2 articles), probe-based qPCR (21 articles), multiplex PCR (32 articles), and droplet digital PCR (2 articles). A variety of downstream approaches were used for amplicon detection and discrimination, including restriction fragment length polymorphism (RFLP; 4 articles), capillary electrophoresis (2 articles), denaturing gradient gel electrophoresis (DGGE) (1 article) and temporal temperature gradient gel electrophoresis (TTGE) (1 article), lateral flow detection, and high-resolution melting analysis (HRMA; 17 articles). Isothermal amplification methods were also represented, most notably loop-mediated isothermal amplification (LAMP; 15 articles) and recombinase polymerase amplification (RPA; 6 articles). In addition, several high-throughput molecular approaches were identified, including next-generation sequencing (NGS; 17 articles), microarrays (8 articles), PCR-quantum dot fluorescence analysis (PCR-QDFA; 1 article), and PCR-electrospray ionization mass spectrometry (PCR-ESI/MS; 4 articles).

The primary target species for identification was *C. albicans* (115 articles), followed by *N. glabratus* (72 articles), *C. parapsilosis* (64 articles), *P. kudriavzevii* (51 articles), and *C. auris* (33 articles). The diagnostic methods used targeted genetic regions, most commonly ITS1/ITS2 (94 articles), 18S rDNA (10 articles), and 28S rDNA (10 articles). *Candida* infection can also be diagnosed in different disease contexts, including oral candidiasis, invasive candidiasis or candidemia, fungal infection due to sepsis, vulvovaginal candidiasis, onychomycosis, otitis externa, or endophthalmitis [28,29,30,31,32,33]. Sample types often reflected the site of infection and included whole blood, vaginal swabs, bronchoalveolar lavage, sputum, and axilla/groin skin swabs [34,35,36,37,38]. The distinction between species identification and fungal infection detection was also made during the article screening. Studies that diagnosed fungal infection were only included if they did so by identifying specific *Candida* species as opposed to pan-fungal amplification.

### 3.1. Quality Assessment of Studies

**Figure 2:**
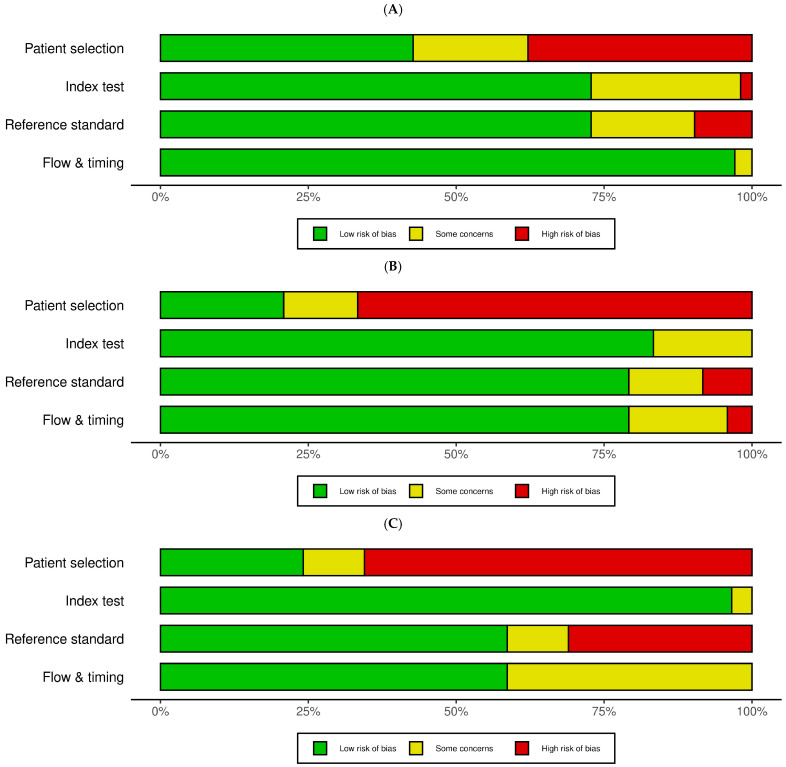
Summary plot for the three different groups of NAAT methods included in this systematic review. (**A**) PCR methods; (**B**) isothermal methods; (**C**) high-throughput methods. Studies were individually assessed for bias and were assigned scores of low risk, some concerns, and high risk of bias for each of the four domains: Patient selection, index test, reference standard, and flow and timing.

The risk of bias was assessed using the QUADAS-2 tool. Traffic light plots with the risk of bias assessed for each study in each of the four domains can be found in the Supplementary information (S2–S4). The summary plots for each group indicating high, some concerns, and low levels of bias risk are displayed in Figure 2.

A. Patient Selection: This domain showed the greatest overall concern, with many studies rated as high risk of bias across the three groups of NAAT methods (38/103 for PCR methods, 17/25 for isothermal methods, and 19/30 for high-throughput methods). The main issues included the frequent use of case-control designs instead of consecutive or random patient sampling (65 articles). For example, Kordalewska et al. 2017 used an unmatched diagnostic case-control (two-gate) design by selecting known *Candida auris* isolates and non-*Candida auris* isolates to evaluate assay performance, rather than consecutively enrolling patients [39]. This can artificially inflate diagnostic accuracy because the positive and negative groups are often very distinct. Numerous studies used pre-selected archived isolates rather than prospectively collected clinical samples, limiting real-world applicability (73 articles). Some studies excluded “difficult” or indeterminate samples without clear justification, introducing selection bias (10 articles). In multiple studies, there was insufficient reporting on how participants or specimens were recruited, leading to unclear risk (53 articles). Last, studies sometimes used pure cultures, stored isolates, or artificially spiked samples, which may not reflect real clinical conditions (23 articles).

B. Index Test: Most studies performed relatively well in this domain, with the majority showing low concern for bias (75/103 for PCR methods; 21/25 for isothermal Methods; 29/30 for high-throughput methods). Strengths included that PCR protocols were standardized and clearly described, and thresholds (Ct values, positivity cutoffs) were usually pre-specified. The main concern overall arose when it was unclear whether the index test was interpreted without knowledge of the reference standard results (35 articles).

C. Reference Standard: The higher risk of bias in the reference standard domain is largely due to the absence of a perfect gold standard for *Candida* identification. In many of the reviewed studies, the reference standard used molecular confirmation methods such as ITS sequencing, microarray-based panels, or other NAATs (63 articles). Concerns were usually low to moderate for ITS sequencing, but high risk was primarily associated with studies using molecular panels in which the reference standard itself incorporated molecular methods too similar to the index test. This can introduce incorporation bias, where the reference standard is not fully independent of the index test. For example, Schabereiter et al. 2007 used ITS rRNA gene sequencing as the reference standard to confirm species identification while evaluating a PCR-based assay [40]. Similarly, Lau et al. 2010 used ITS sequencing as the reference standard to evaluate a real-time PCR assay for Candida species detection [41]. Last, a few studies lacked sufficient detail on how species confirmation was performed, leading to unclear risk, represented as some concerns in the plots (23 articles).

D. Flow and Timing: Overall, there was a low risk of bias and minimal delay between index and reference testing. Usually, all patients were included in the study and received the same reference standard. However, 12 of the 30 high-throughput studies were judged as having some concerns, primarily because of insufficient reporting of participant flow. For example, in Banik et al. 2024, it was unclear whether all enrolled samples were included in the final analysis [42]. Most studies showed little to no delay between index and reference testing.

#### 3.1.1. Conventional PCR (Polymerase Chain Reaction)

**Figure 3:**
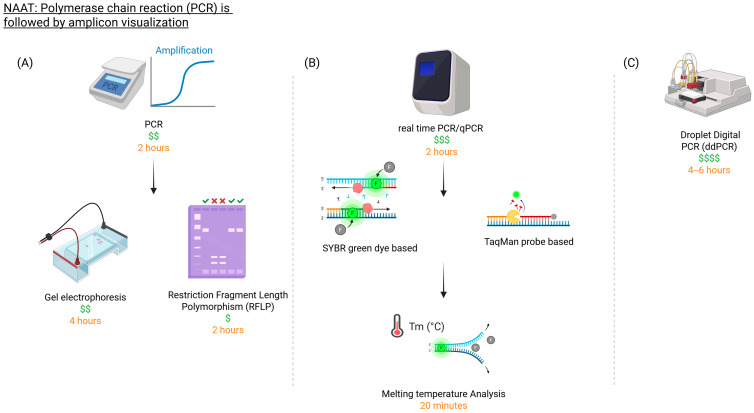
Summary of PCR-based nucleic acid amplification tests (NAAT) used for *Candida* species identification followed by amplicon visualization. (**A**) Polymerase chain reaction (PCR) methods and amplicon visualization using gel electrophoresis and restriction fragment length polymorphism (RFLP) analysis. (**B**) Real-time PCR/qPCR that is SYBR^®^ Green dye-based or TaqMan^®^ probe-based. (**C**) Droplet digital PCR (ddPCR). Created in BioRender (BioRender Inc., Toronto, ON, Canada). Tharmalingam, S. (2026). https://BioRender.com/.hjesrul (accessed on 14 May 2026).

PCR is a widely used nucleic acid amplification technique that can detect very small amounts of DNA by amplifying the target into millions of copies. Simplex PCR amplifies a single target. PCR offers high specificity when primers are accurately designed for the target organism. After amplification, products are commonly visualized using gel electrophoresis, where amplicon size is compared to a DNA ladder. However, evolutionary variability in intron size within strains could produce multiple gel band lengths, leading to ambiguous interpretation. Despite the potential advantages, PCR-based methods are limited by the need for high-quality nucleic acid extraction, susceptibility to sample loss during purification, and reliance on expensive equipment and skilled personnel. These requirements may limit their use in point-of-care and resource-limited settings [38]. Figure 3 presents a summary of the PCR-based methods.

There were 17 articles that primarily used standard PCR for *Candida* species identification (Table 1) [23,33,34,38,39,43,44,45,46,47,48,49,50,51,52,53,54]. Martinez et al. (2010) amplified the intron region of the *RPS0* gene for eight *Candida* species using pan-fungal primers, and then identified species based on amplicon size [44]. The reported LoD of approximately 10^3^ cells/mL, despite high specificity (100%), could hamper clinical utility. In contrast, Bineshian et al. (2015) employed species-specific primers to target the *MP65* gene for *C. albicans*, *N. glabratus*, and *C. parapsilosis*, after DNA was extracted with a phenol-chloroform protocol [48]. The assay demonstrated improved sensitivity, with a LoD of ~50 yeast cells/reaction.

García-Salazar et al. (2022) developed a simplex PCR assay targeting the 18S-28S rDNA region of eight clinically relevant *Candida* species using validated CandF and CandR primers [51]. The sensitivity (~74%) was lower than desired and corresponded to limits of detection of 10 pg/µL of DNA or 10^3^ yeast cells/mL. Although such PCR assays may be considered cost-effective and relatively simple to run, the method can be improved upon as the qualitative nature does not enable fungal load quantification. Methods like RT-qPCR would be useful instead for burden assessment.

Detecting *C. auris* is also a pressing objective found in this review. Using PCR, Kordalewska et al. (2017) detected *C. auris* with an LoD of 10 CFU/mL and 100% sensitivity and specificity for the species-specific assay, while the LoD was 10^3^ CFU/mL for the assay detecting *C. auris*-related species [39]. Ruiz-Gaitan et al. (2018) used PCR to target *C. auris* species-specific genes encoding glycosylphosphatidylinositol (GPI) proteins with no paralogs in other *Candida* species, resulting in high specificity (100%) and no false positives [50]. Additionally, two primer pairs targeting the same region were utilized, so reliability was increased with the redundancy. This could be a useful method in low-cost settings.

#### 3.1.2. LATE-PCR for *Candida* Species Identification

Linear-after-the-exponential PCR (LATE-PCR) is an asymmetric technique that uses unequal primer concentrations, with one in excess and one limiting primer [55]. Early cycles produce exponential double-stranded amplification, followed by linear generation of single-stranded DNA once the limiting primer is depleted. LATE-PCR remains infrequently used in diagnostic studies, potentially because of the costs and time associated with a more complex primer design and optimization. Only one article was identified in this review by Gentile et al. (2013) (Table 1) [53]. The assay had excellent 100% sensitivity for the species identified, including *C. albicans* (32/32), *C. dubliniensis* (1/1), *N. glabratus* (8/8), and *C. parapsilosis* (4/4). The measure of specificity was lacking and would need to be completed to make use of this method [53].

#### 3.1.3. Nested and Semi-Nested PCR Used for *Candida* Species Identification

Nested and semi-nested PCR designs enhance sensitivity and specificity through sequential primer sets that reduce non-specific amplification. Outer primers are followed by inner primers designed to target a region within the initial amplicon. Semi-nested PCR employs one inner and one outer primer in the second reaction [56].

Nine articles used a nested PCR design (Table 2) [24,57,58,59,60,61,62,63,64]. Landlinger et al. (2009) used ITS2-semi-nested PCR coupled with Luminex bead-based detection, in which biotinylated amplicons hybridize to probe-coated xMAP beads (Luminex Corporation, Austin, TX, USA) [64]. Nine *Candida* species were targeted in addition to other fungal species. One sample of *C. dubliniensis* was confirmed with the Luminex assay, whereas standard blood culture methods identified it as *C. albicans*. Gosiewski et al. (2014) used outer primers that amplified the 18S rDNA region with a set of internal primers and TaqMan^TM^ probes (Thermo Fisher Scientific, Foster City, CA, USA) to identify *C. albicans* [60]. The nested PCR had greater sensitivity (10^1^ CFU/mL) than the multiplexed second amplification reaction (9.9 × 10^2^ ± 3.4 × 10^3^ CFU/mL).

FilmArray BCID2 is a nested PCR-based assay commercialized as a fungal infection detection kit (bioMérieux, Marcy-l’Étoile, France) that can be used to process positive blood culture samples [62]. The panel includes six *Candida* species, with *C. auris* added in the updated BCID2 version, which enhanced this test’s clinical utility. After cell lysis and DNA extraction, the assay has a two-stage nested PCR workflow, and detection was achieved by real-time qPCR using a fluorescent dye. The turnaround time for results was approximately 3.5 h, which is shorter than conventional culture-based identification, which typically requires days to grow fungal cultures. However, detection is restricted to on-panel targets, limiting usability for off-panel or mixed fungal infections. The BCID2 kit was consistent with conventional blood culturing results, making it a diagnostic technique with a positive percent agreement of 97% for the organisms tested.

#### 3.1.4. RFLP Used for *Candida* Species Identification

For Restriction Fragment Length Polymorphism (RFLP) analysis, the target gene is PCR amplified, and then one or more restriction endonucleases are added to the reaction mixture [65]. The resulting amplicons are visualized using gel electrophoresis. Each target species produces a unique band pattern depending on the number of restriction sites present in the gene sequence [65]. The gene chosen for this technique needs to have very low intra-species variability. Otherwise, though the PCR-RFLP method is less expensive and easier to complete, the results may still need to be verified with sequencing.

Four articles used this PCR-RFLP method (Table 3) [28,66,67,68]. A study in Iran identified all but 6 of 204 blood culture samples that tested yeast-positive for *C. albicans*, *C. parapsilosis*, *N. glabratus*, *P. kudriavzevii* and *C. tropicalis* [67]. The remaining samples were identified using ITS sequencing. Restriction enzyme digestion was performed with MspI (Thermo Fisher Scientific, Foster City, CA, USA) and targeted the ITS1-ITS2 region (amplified with pan-fungal (ITS1 and ITS4) primers). Alternatively, Szemiako et al. (2017) targeted the homocitrate synthase gene with fluorescent labeled primers bound to the PCR amplicon [66]. All 75 clinical isolates were correctly identified compared to the traditional phenotypic method of CHROMAgar ^®^ *Candida* (CHROMagar Paris, Paris, France) [66].

Marcos-Arias et al. (2020) focused on *C. parapsilosis* detection and its similar species *C. orthopsilosis* and *C. metapsilosis* [68]. The targets for restriction enzyme digestion were the *SADH* and *FKS1* genes, and these results were compared to the sequenced D1/D2 rDNA control [68]. For *C. parapsilosis*, sensitivity was 99% for *SADH* and 97% for *FKS1*, while the specificity for *FKS1* was higher than that for *SADH* (100% vs. 78%). For detection of *C. orthopsilosis*, both sensitivity and specificity were identical for *SADH* and *FKS1* (77% and 99%, respectively). *C. metapsilosis* specificity was also 100% for both genes, while *FKS1* had a higher sensitivity (40% vs. 0%). One drawback was that the *SADH* gene could not correctly distinguish between 10 samples of *C. orthopsilosis* and *C. metapsilosis* [68]. This could be due to allelic variation within species and loss of restriction endonuclease cutting sites. More recently, Jabrodini et al. (2024) used PCR-RFLP for detecting *C. albicans*, *C. parapsilosis* and *C. tropicalis* [28]. Sensitivity was below 90% (88.5%), but the specificity was 100% [28].

#### 3.1.5. PCR Amplicon Visualization: Capillary Electrophoresis

Capillary electrophoresis (CE) separates DNA fragments within a thin capillary tube filled with a polymer matrix (Table 1) and detects fluorescently labeled amplicons by a laser or photodetector [49]. Compared to traditional gel electrophoresis, this method offers faster run times, higher resolution, and improved quantification. However, the cost is high for the CE instrument along with the reagents, which may limit the use of CE in a resource-limited laboratory setting [49,69].

#### 3.1.6. PCR Amplicon Visualization: DGGE and TTGE

Denaturing Gradient Gel Electrophoresis (DGGE) and Temporal Temperature Gradient Gel Electrophoresis (TTGE) are techniques used to separate DNA amplicons of identical length but different nucleotide sequences. These methods exploit sequence-dependent differences in DNA melting behavior, causing partially denatured DNA fragments to migrate at different rates through a gel and generate characteristic banding patterns for species identification [34]. To prevent complete strand separation during the partial denaturation process, a GC clamp is used in the primer design to stabilize the DNA structure [34]. DGGE is completed with a polyacrylamide gel with a chemical denaturing gradient (e.g., urea and formamide), whereas gradual denaturation occurs through increasing temperature in TTGE [34].

Mohammadi et al. (2015) used conventional PCR to amplify two different regions (either the D1 region of the 26–28s rDNA gene or the ITS2 region) of the selected *Candida* species (*C. albicans*, *N. glabratus*, *C. tropicalis*, and *C. orthopsilosis*) [34]. The results were visualized with the use of either DGGE or TTGE. The results were better for the D1 region than for the ITS2 region, in terms of clear, specific PCR products that could be differentiated between species. TTGE was easier to complete despite DGGE having better band resolution.

#### 3.1.7. PCR Amplicon Visualization: Lateral Flow Detection

Lateral flow detection is a post-amplification method used for rapid visualization of PCR products. This technique requires primers that are labeled with biotin and fluorescein isothiocyanate (FITC) at opposite ends of the amplicon, which allows for a lateral flow transfer strip setup [70]. A species-specific signal is used for identification, which is produced through amplicon interaction with anti-biotin ligands, while the control line typically has anti-mouse and anti-FITC antibodies bound to the strip to verify assay function [70]. Srichaiyapol et al. (2025) used the ITS2 region for *C. albicans*-specific primers and then a lateral flow transfer for the species identification readout (Table 1) [54]. There were two options for DNA input: either DNA extraction from a blood sample or taking a single clonal colony from a plate.

### 3.2. qPCR/RT-PCR Methods Used for Candida Species Identification

Quantitative PCR (qPCR), also known as real-time PCR (RT-PCR), continuously monitors DNA amplification as it occurs, rather than only at the end of the reaction. The signal from fluorescent dyes that intercalate with double-stranded DNA, or sequence-specific fluorescent probes, increases in fluorescence intensity proportionally with the amount of amplified DNA. The amount of the initial DNA template present is based on the threshold cycle where maximum amplification was reached. The high sensitivity and specificity possible with RT-PCR can be used to distinguish between closely related species and estimate pathogen load, even for low-abundance targets. Its real-time output enables faster turnaround times compared to conventional PCR methods. The risk of contamination associated with post-PCR processing is also eliminated.

#### 3.2.1. Real-Time PCR with SYBR Green Dye

Two articles used dye-based qPCR for species identification (Table 4) [71,72]. Felix et al. (2023) detected *C. albicans*, *N. glabratus*, *C. parapsilosis* (including the *C. parapsilosis* complex), *C. tropicalis*, and *P. kudriavzevii* in blood samples [72]. The method had good concordance compared to culture and was able to detect 4 infections consisting of more than one microbe, which culture missed. In Shuping et al. (2023), SYBR PrimeScript^TM^ RT-PCR (Thermo Fisher Scientific, Eugene, OR, USA) had a lower sensitivity of 44% when identifying *C. auris* during an outbreak in South Africa [71].

#### 3.2.2. Real-Time PCR with Probes

In probe-based real-time PCR, a sequence-specific probe binds to the complementary target DNA sequence located between the forward and reverse primers. The probe is also modified with a fluorescent reporter molecule on one end and a quencher molecule on the other end. During the extension phase of PCR, the 5′-3′ exonuclease activity of *Taq* polymerase results in probe cleavage, which separates the reporter from the quencher. The resulting increase in fluorescence is proportional to the amount of amplified PCR product and can be detected.

Nineteen articles used a PCR technique in addition to DNA probes (Table 5) [29,36,42,73,74,75,76,77,78,79,80,81,82,83,84,85,86,87,88]. McMullan et al. (2008) applied TaqMan^TM^-based RT-PCR (Thermo Fisher Scientific, Foster City, CA, USA), developing three assays: one targeting fluconazole-susceptible species (*C. albicans*, *C. parapsilosis*, *C. tropicalis*, and *C. dubliniensis*) and separate assays for the resistant species *N. glabratus* and *P. kudriavzevii* [73]. Testing over 500 serum samples from 157 patients, they reported high specificity (100%) and sensitivity (87%).

Martínez-Murcia et al. (2018) used GPS Monodosedtec qPCR (Mobidiag, Helsinki, Finland) probes to identify *C. auris*, with a detection limit of 5–10 DNA copies/reaction [78]. Leach et al. (2019) developed an RT-PCR assay for *C. auris* using the BD MAX platform (Becton Dickinson, Franklin Lakes, NJ, USA) and an optimized 20-min DNA extraction [79]. This assay showed very high analytical sensitivity (LoD of 1 CFU/reaction) and showed no cross-reactivity with other *Candida* species, improving upon an earlier version [77]. Ahmad et al. (2019) also targeted the ITS2 region of *C. auris* with TaqMan^TM^ probes, reporting high sensitivity (93.6%) and specificity (97.2%) [80].

The commercial kit AurisID^®^ (OLM Diagnostics’, Oxford, UK) tested by Mulet-Bayona et al. (2021) could detect *C. auris* from surveillance swab samples [84]. It had high sensitivity (96.6%) and 100% specificity [84]. The LoD of 500 CFU/mL translates to about 1 CFU per reaction, meaning this kit could detect low fungal load infections. One benefit is that Mulet Bayona et al. (2021) used a protocol that did not require prior DNA extraction [84]. While this saves time, it could compromise sensitivity, especially in cases of low fungal load or if PCR inhibitors are in the sample medium. Sattler et al. (2021) compared AurisID^®^ with Fungiplex^®^ (Bruker Daltonics, Bremen, Germany) [83]. AurisID^®^ demonstrated higher sensitivity, detecting as low as 1 genome copy/reaction, compared with 9 copies/reaction for Fungiplex^®^. However, AurisID^®^ showed some cross-reactivity at high DNA input, as there was reduced specificity with closely related species, while Fungiplex^®^ was 100% specific. Overall, both kits could be used for clinical *C. auris* detection, with AurisID^®^ providing greater sensitivity and Fungiplex^®^ yielding slightly higher specificity [83].

Leonhard et al. (2024) similarly developed a TaqMan^TM^ PCR assay with excellent 100% sensitivity and specificity, and a low LoD of 4 cells per reaction [81]. Next, Dehesa-Garcia et al. (2025) validated the VIASURE assay (Certest Biotec, Zaragoza, Spain) for identification of *C. auris* using FAM-labeled probes, achieving high sensitivity (98%) and 100% specificity [82]. Nhan et al. (2025) detected *C. auris* with 100% specificity and a very low analytical sensitivity of 1.95 log CFU/mL from nasal swabs [88].

Rosa et al. (2023) demonstrated the usefulness of screening for *C. auris* with an in-house PCR protocol, and on average it reduced the time to a result from around 11 days to 2 days [86]. They used a qPCR platform targeting the ITS2 region, which was then identified with a fluorescent probe signal. They estimated substantial savings from completing this screening faster and making it more accessible in the clinic [86]. Banik et al. (2024) tested the diagnostic performance of their developed cartridge-based RT-PCR assay called CaurisSurV, which ran on the GeneXpert instrument (Cepheid, Sunnyvale, CA, USA) [42]. The design detected *C. auris* from skin swabs based on molecular beacon probes. The LoD was 10.5–14.8 CFU/mL and specificity was 100%. Franco et al. (2024) also used an RT-PCR assay for identification of *C. auris* but with ITS2 primers (DiaSorin Molecular, Simplexa^TM^, Hilden, Germany) and showed a very low LoD of 1–2 CFU/mL [36]. Ramirez et al. (2023) used the same primers, yet the LoD was substantially higher at 266 CFU/µL [87]. Ibrahim et al. (2023) detected *C. auris* by targeting a GPI (glycosyl-phosphatidylinositol) protein-encoding gene rather than the typical ITS2 region [85]. The LoD was 13 CFU/mL and specificity was 100%.

#### 3.2.3. ELISA Assay Combining Probes and RT-PCR

For an ELISA assay, PCR incorporates a biotin label into the amplicon via a primer. A digoxigenin (DIG)-labeled probe then binds the target DNA sequence. The resulting biotin- and DIG-labeled amplicon attaches to streptavidin on an ELISA plate and is detected using an anti-DIG antibody with a colorimetric substrate [89]. One study combined initial DNA amplification from blood samples with an ELISA-based hybridization assay. species-specific probes targeted *C. tropicalis* and *P. kudriavzevii* [74]. The assay showed a sensitivity of 84.6% and a specificity of approximately 93%. In immunosuppressed patients monitored over six months, this PCR-ELISA detected invasive fungal infection an average of 21 days earlier than diagnosis based on clinical signs (mean 39 days). While useful for early detection and ruling out infection, the method was recommended as an addition to conventional diagnostics rather than a standalone test.

#### 3.2.4. Multiplex PCR/Multiplex qPCR Methods Used for *Candida* Species Identification

Multiplex PCR allows simultaneous amplification of multiple target regions in a single reaction using multiple primer sets and probe technology. There were 32 articles in the Multiplex PCR/RT-PCR category (Table 6) [30,31,32,69,90,91,92,93,94,95,96,97,98,99,100,101,102,103,104,105,106,107,108,109,110,111,112,113,114,115,116,117,118]. Carvalho et al. (2007) combined ITS primers to PCR amplify a control region in addition to complexing the reaction with 8 species-specific primers targeting *C. albicans*, *N. glabratus*, *C. parapsilosis*, *C. tropicalis*, *P. kudriavzevii*, *C. guilliermondii*, *C. lusitaniae* and *C. dubliniensis* [91]. Innings et al. (2007) achieved detection limits of 1–10 genome copies for *C. albicans*, *C. guilliermondii*, *N. glabratus*, and *P. kudriavzevii* using probes targeting the RNase P RNA (RPR1) gene [105]. Vahidnia et al. (2015) identified *C. albicans* and *C. parapsilosis* toenail fungal infection with a multiplex PCR that delivered the result within 24 h compared to 4 weeks with culturing methods [30]. There was also 100% sensitivity and 99.32% specificity [30].

Balada-Llasat et al. (2012) tested the Luminex xTag^®^ Fungal Assay (Luminex Corporation, Austin, TX, USA) for 7 *Candida* species [92]. The *C. albicans* gene target was *HWP1*, and RNase P was used for the other six: *N. glabratus*, *C. parapsilosis*, *C. tropicalis*, *P. kudriavzevii*, *C. lusitaniae*, *C. guilliermondii* [92]. The different PCR amplicons were detected in a bead solution bound to probes. By counting the beads signaling each species, assay sensitivity was 100%, and specificity was 99% [92]. For fungal endophthalmitis, Sugita et al. (2012) used qPCR targeting the 18S rRNA gene for broad detection, followed by multiplexing 6 species-specific TaqMan^TM^ probes (Thermo Fisher Scientific, Foster City, CA, USA) [106]. Sampath et al. (2017) detected 4 *Candida* species using a universal primer and then species-specific primers across the ITS region [95]. They considered at-risk amounts for candidiasis at >600 CFU/mL [95]. Another commercial kit tested was Microbscan (bioMérieux, Marcy-l’Étoile, France) which detected *C. albicans*, *N. glabratus*, *P. kudriavzevii*, *C. orthopsilosis*, *C. parapsilosis* and *C. tropicalis* [96].

Monstein et al. (2014) tested the Seegene Seeplex^®^ STI Master Panel 3 multiplex PCR (Seegene Inc., Seoul, South Korea), which also used capillary gel electrophoresis for the identification of six different *Candida* species (*N. glabratus*, *C. tropicalis*, *C. parapsilosis*, *P. kudriavzevii*, *C. albicans* and *C. dubliniensis*) [69]. They found that, compared to ITS sequencing, the Seeplex^®^ assay had relatively good concordance, and suggested this method could be used for screening. One study had quite low sensitivity and specificity (67.4% and 41.9%, respectively) for *C. albicans* and *C. parapsilosis* detection in mammary candidiasis infections [110]. The MycoReal *Candida* commercial kit (Sacace Biotechnologies, Milan, Italy) was tested and was deemed to not have diagnostic value compared to traditional cultures [110].

Fortún et al. (2014) validated a multiplex RT-PCR assay on clinical blood and serum samples targeting major species causing invasive candidiasis in ICU patients (*C. albicans*, *C. parapsilosis*, *C. tropicalis*, *N. glabratus*, *P. kudriavzevii*, and *C. guilliermondii*) [107]. Species were detected with fluorescent molecular beacon probes, and reactions were split into two tubes to identify three species per tube. A key limitation was that PCR positivity could not distinguish invasive infection from heavy colonization, where about 65% of patients showed significant colonization without confirmed invasive candidiasis. Fukumoto et al. (2015) developed primer-probe sets for broad bacterial and fungal detection, including *C. albicans*, in lung autopsy samples [109], while Masha et al. (2018) evaluated TaqMan^TM^ Array Cards for identifying urogenital pathogens, including *C. albicans* [111].

Nakano et al. (2017) included *N. glabrata* and *P. kudriavzevii* among 24 common ocular pathogens for identification using multiplex solid-phase strip PCR (STRIP PCR) [31]. The design consisted of a 12-well strip tube, and each well was coated with immobilized forward and reverse primers and probes designated for identifying 1–3 pathogens per tube [31]. The amplification was measured with a LightCycler qPCR machine (Roche Applied Science, Penzberg, Germany). It had similar sensitivity compared to conventional PCR designs, and the ability to multiplex so many target species is an advantage as well.

Arastehfar et al. (2018) utilized endpoint tetraplex PCR for *C. auris* and three closely related species (*C. haemulonii*, *C. duobushaemulonii* and *C. pseudohaemulonii*) [118]. Arastehfar et al. (2019) tested the YEAST PANEL multiplex PCR for seven major pathogenic and seven minor pathogenic *Candida* species [98]. The Fungiplex^®^ kit was used to test blood samples for invasive *Candida* infection (candidemia), with high specificity (94%) and 100% sensitivity, but the PPV was 63% [97]. Amor et al. (2024) described the Vircell Vaginal Panel (Vircell/Virotec, Granada, Spain), which identified multiple *Candida* species and incorporated an endogenous RNase P control to assess sample quality and PCR inhibition [32].

The DermaGenius kit (Eurofins Genomics/DiaTech, Jesi, Italy) is an RT-PCR assay used to detect *C. albicans* and various dermatophyte pathogens in nail samples [113]. The diagnostic measures were comparable to culturing and histology, and it could improve the time to result [113]. Ndiaye et al. (2022) also tested the DermaGenius^®^ kit with slightly improved sensitivity (89.3% vs. 80%) and specificity (75.3% vs. 74.4%) [114]. Aboutalebian et al. (2021) used multiplex PCR to diagnose the fungal ear infection Otitis externa, which can be caused by *C. albicans* [100]. They also tested Yeast plex (Mobidiag, Helsinki, Finland) using species-specific primers and gel electrophoresis [102]. In the latter study, new primers were designed to limit the amplicon size to 500 bp to improve sensitivity compared to the previous iteration of this assay that used 700–800 bp [100]. Li et al. (2023) developed a multiplex PCR to identify *C. albicans* and other sepsis pathogens, using immobilized probes on a biomembrane chip to hybridize to PCR amplicons [103]. The AccuPower^®^ STI4CPlex Real-Time PCR Kit (Bioneer, Daejeon, South Korea) was tested for its detection of *C. albicans* among 3 other non-*Candida* pathogens, and the LoD was ~148 copies/mL [115].

Numerous commercial qPCR assays have been evaluated for identifying *Candida* species in fungal sepsis, where bloodstream infection triggers a systemic inflammatory response [94]. Elges et al. (2017) evaluated SeptiFast^®^ (Roche Molecular Systems, Pleasanton, CA, USA), a multiplex RT-PCR assay for pathogen monitoring in hematological patients following allogeneic stem cell transplantation [94]. *C. albicans*, *N. glabrata*, *C. tropicalis*, *C. parapsilosis*, and *P. kudriavzevii* were identified in bile samples, albeit in a small cohort (26 patients) [102]. Although SeptiFast^®^ could identify both bacterial and fungal sepsis pathogens, it is no longer commercially available [102]. Camp et al. (2024) compared SeptiFast^®^ with the T2MR (T2*Candida*) panel (T2 Biosystems, Lexington, MA, USA) and found comparable diagnostic performance for candidemia [104]. SeptiFast^®^ showed slightly higher sensitivity for proven or probable candidemia (81.1%) than T2*Candida* (73.0%), while sensitivities for proven cases alone were similar (68.2% vs. 63.6%). Overall, the authors concluded that T2*Candida* is a suitable replacement for the discontinued SeptiFast^®^ as a rapid molecular diagnostic tool.

Schmitt et al. (2014) used multiplex qPCR and target-specific oligonucleotide probes to detect STI pathogens including *C. albicans*, *N. glabratus* and *P. kudriavzevii* [108]. This specific mRT-PCR generated biotinylated amplicons and was followed by a bead-based xMAP hybridization assay [108]. The probes were bound to Luminex beads (Luminex Corporation, Austin, TX, USA), so the cost and resources required for this extra step may limit the usefulness of this assay in the clinic. Bui et al. (2023) also detected STI pathogens including *C. albicans* with the use of double-quenched TaqMan^TM^ probes for improved sensitivity [116]. They used a large clinical sample set of 535 vaginal swab samples, and the gene target was RPR1 (RNase P RNA component 1). Garcia-Salazar et al. (2025) developed Cand-PCR, which can identify eight clinically relevant *Candida* species involved in the diagnosis of vulvovaginal candidiasis (VVC) [117]. The sensitivity was lower than other methods at 65%. However, it had excellent specificity (100%) and predictive values (PPV = 100%, NPV = 91%), which would be useful in accurate diagnosis.

#### 3.2.5. HRMA Used for *Candida* Species Identification

High-resolution melting analysis (HRMA) requires qPCR to first amplify DNA with a fluorescent dye (e.g., EvaGreen, Bio-Rad Laboratories, Hercules, CA, USA) that binds to double-stranded DNA. As the temperature is increased, the DNA denatures, releasing the dye and reducing fluorescence. The signal is recorded as a melting curve, and each *Candida* species shows a distinct melting peak at a specific melting temperature (T^m^) which can be used for species identification [119]. 17 studies using melt curve analysis for *Candida* species identification are cited (Table 7) [40,41,119,120,121,122,123,124,125,126,127,128,129,130,131,132,133]. Bu et al. (2005) distinguished between 3 *Candida* species (*C. albicans*, *C. tropicalis* and *P. kudriavzevii*) using a multiplex RT-PCR design followed by HRMA [120]. There was good sensitivity with a LoD at 0.1 pg fungal genomic DNA, or about 3 fungal cells, and no false negatives, resulting in excellent sensitivity.

Klingspor et al. (2006) identified *C. albicans*, *N. glabratus*, *C. tropicalis*, *C. parapsilosis*, and *P. kudriavzevii* with species-specific probes that hybridize to the 18S rRNA PCR amplicon followed by melting curve analysis [121]. Fricke et al. (2010) identified 5 key *Candida* species (*C. albicans*, *C. parapsilosis*, *N. glabratus*, *C. tropicalis* and *C. dubliniensis*) down to 2 genome equivalents per reaction [122]. Lau et al. (2010) found a lower sensitivity of 75%, and a specificity of 97% using this method [41]. Ashrafi et al. (2015) demonstrated high sensitivity as low as 1 yeast cell (~10 fg) of DNA [125]. DNA was amplified by common primers, and then a fluorescent resonance energy transfer (FRET) probe detected the fluorescent signal of PCR amplification. Guo et al. (2016) targeted the 5.8S rRNA gene first with primers and then species-specific probes that bound to the PCR amplicon from blood cultures [76]. At 10 CFU/mL, the sensitivity for *C. albicans*, *C. parapsilosis*, *C. tropicalis*, and *P. kudriavzevii* was 100%, while for *N. glabrata* the sensitivity was lower at around 75%. This probe would need refinement to improve the utility of this assay, especially for fluconazole-resistant species. Zhang et al. (2016) identified eight species, and the LoD was as low as 10 fg of DNA [126]. Fidler et al. (2018) displayed the utility of this method by developing and testing the CanTub-simplex PCR, where sensitivity was down to 0.2–2 genome equivalents per reaction [127].

Walchak et al. (2020) studied melting curve differences to distinguish between *C. auris* and its closely related species in clinical axilla/groin swab samples with good specificity of 100% (sensitivity was ≥100 CFU/mL) [130]. Alvarado et al. (2021) also detected *C. auris* and related species in addition to *C. lusitaniae* and *C. albicans* by targeting GPI protein-encoding sequences with 100% specificity testing [131].

### 3.3. Droplet Digital PCR (ddPCR) Used for Candida Species Identification

DdPCR partitions a PCR reaction into thousands of nanoliter droplets, each containing a fluorescent probe. After amplification, fluorescent droplets indicate target DNA, enabling single-copy detection [134]. The positive and negative droplets are counted to generate highly sensitive quantification that can be useful for measuring fungal load and multiplex detection. Both articles using ddPCR [134,135] had high specificity at around 5 cells/DNA copies per reaction when identifying *C. albicans* (Table 8).

## 4. Isothermal Amplification NAAT Methods: LAMP, RPA and RAA

**Figure 4:**
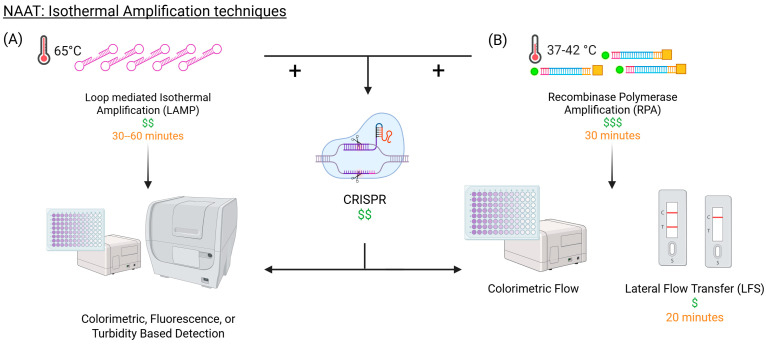
Summary figure of different isothermal nucleic acid amplification tests (NAAT) used for *Candida* species identification. Isothermal temperature amplification methods include (**A**) loop-mediated isothermal amplification (LAMP) and (**B**) recombinase polymerase amplification (RPA). These two methods can be coupled with CRISPR to improve sensitivity and specificity. Reaction amplicons can be visualized using colorimetric, fluorescence, or turbidity-based detection, or lateral flow transfer. Created in BioRender (BioRender Inc., Toronto, ON, Canada). Tharmalingam, S. (2026). https://BioRender.com/0obyq35 (accessed on 14 May 2026).

Isothermal NAATs offer an alternative to PCR-based methods by eliminating the need for thermal cycling. These assays utilize DNA polymerases with strand displacement activity to enable nucleic acid amplification at a constant temperature. This reduces equipment complexity and supports rapid, point-of-care diagnostics. Key platforms include loop-mediated isothermal amplification (LAMP), recombinase polymerase amplification (RPA), recombinase-aided amplification (RAA), and related rapid methods (Figure 4).

### 4.1. LAMP Used for Candida Species Identification

LAMP uses *Bst* DNA polymerase derived from the bacterium *Geobacillus stearothermophilus* to amplify DNA at a constant temperature of 65 °C [136]. The LAMP reaction employs 4–6 primers that span the target sequence in a design that amplifies large amounts of DNA. The strand displacement activity of the polymerase results in the production of concatemeric, dumbbell-shaped amplicons. The rapid amplification enabled by LAMP requires simpler, low-cost equipment than PCR, making LAMP potentially more suitable for point-of-care and resource-limited settings [137]. However, the use of multiple primers increases the risk of primer-primer interactions like dimers, thus producing false positives. In addition, strict laboratory controls are needed when LAMP is applied because the high amplification efficiency of the reaction increases the risk of aerosolized amplicon contamination.

A total of 13 articles were cited using the LAMP technique (Table 9) [18,138,139,140,141,142,143,144,145,146,147,148,149]. Several of these studies have developed LAMP assays for specific detection of *C. albicans* [138,139,140,141]. Noguchi et al. (2017) tested oral exfoliative cytology samples and applied a commercial “Loopamp Specified Microorganism Fungi *Candida albicans* Detection Kit” (Eiken Chemical, Tokyo, Japan) after DNA extraction [138]. They achieved a LoD of 1 pg/reaction. Fallahi et al. (2020) designed primers targeting the ITS2 region and reached a LoD of 10 fg along with confirmed primer specificity against multiple species [139]. Wang et al. (2022) combined LAMP with a lateral flow biosensor (LFB) assay, where nanoparticles binding to the test line antibodies indicate amplification alongside a positive control [140]. The process, including DNA extraction and a 40-min isothermal LAMP reaction, was completed in 85 min. Specificity was 100%, but the required lateral flow transfer and open caps increased the risk of aerosol contamination from the highly amplified LAMP products.

Li et al. (2023) developed a LAMP assay to identify VVC-causing species like *C. albicans* [143]. Compared with the reference method of ITS-region PCR followed by sequencing, this LAMP assay was more specific for *C. albicans* (100% vs. 90.4%). Pre-treatment of the fungal cells included lyticase, bead-beating, and thermal denaturation to break the cell wall and membranes and make DNA accessible to primers. Specificity was 100% for all species, while sensitivity varied (*C. albicans* 94%, *N. glabratus* 100%, and *C. tropicalis* 80%). Jin et al. (2023) described an alternate LAMP-based rapid sample processing (RPT) system for detecting *C. albicans*, *N. glabratus*, *C. parapsilosis*, and *C. tropicalis* with high sensitivity (~2 CFU/reaction) [144]. Sample prep also used a lysis buffer solution of sorbitol and EDTA in PBS, along with lyticase and glass beads to lyse fungal cells. The extracted DNA was transferred to a microfluidic chip with 24 reaction chambers containing LAMP reagents. A fluorescent dye bound to the resulting amplicons signals the detection of specific species based on the uniquely designed primer set in each chamber.

Yahaya et al. (2024) developed a LAMP assay targeting the ITS region for specific detection of *N. glabratus*, with a time to result within 45 min [145]. The assay was highly sensitive, detecting 2.25 × 10^0^ copies/µL compared to 2.25 × 10^3^ copies/µL for the reference PCR, and showed 100% specificity against eight closely related pathogenic *Candida* species including *C. albicans* and *C. tropicalis*. Wang et al. (2024) developed an endonuclease-mediated real-time LAMP (ERT-LAMP-CA) protocol for testing sputum samples without the need for open caps, which can introduce contamination into the surrounding lab environment [147]. The method incorporated a restriction endonuclease recognition sequence, fluorescent reporter, and quencher into the FIP primer design. When the endonuclease cleaves the sequence at the restriction enzyme cut site, the quenched fluorophore is released and can be detected by an RT-PCR instrument. This approach achieved a high sensitivity of 500 ag/µL.

The Eazyplex^®^ *Candida* ID assay (Optivagenics, Lörrach, Germany) is a commercial research kit that identifies six *Candida* species (*C. albicans*, *N. glabratus*, *P. kudriavzevii*, *C. tropicalis*, *C. parapsilosis*, and *C. auris*) [146]. LAMP primers targeted the following species-specific genes: *COX1*, ITS rDNA, *COX1*, *COX3*, *COX3*, and *NAD5*, respectively. DNA was extracted using heat, magnetic beads, and lysis buffer, then added to test strips containing lyophilized LAMP reagents. The reaction is monitored by measuring the fluorescence signal via the Genie ^®^HT instrument (Optivagenics, Lörrach, Germany), which is specifically designed for high-throughput diagnostics of isothermal LAMP assays. Sensitivity and specificity varied by species: *C. albicans* (93.9%/99.3%), *N. glabratus* (89.1%/100%), *P. kudriavzevii* and *C. tropicalis* (100%/100%), and *C. parapsilosis* (100%/99.4%) [146]. Hernández Felices et al. (2024) tested this Eazyplex^®^ system specifically for *C. auris* with 91.8% sensitivity and 98.8% specificity [18]. Yamamoto et al. (2024) evaluated an alternate commercially available test named LAMPAuris (Eiken Chemical, Tokyo, Japan) for detecting *C. auris* [148]. They found high specificity at 97–100% while sensitivity was more variable (66–86%) when compared to the reference method of qPCR.

### 4.2. Closed Dumbbell-Mediated Isothermal Amplification

An augmentation of the LAMP technique is closed dumbbell-mediated isothermal amplification (CDA). Numerous primers are designed, including 2 forward, 2 reverse, middle, and loop primer sequences [149]. Closed dumbbell shapes are created as opposed to the loop concatemer structures formed in LAMP. Hydroxy naphthol blue (HNB) can be used to visualize the amplification results with a color change, which provides ease of use in a point-of-care setting. CDA was used to detect *C. albicans* by targeting the ITS2 region (Table 9) [149]. High specificity was determined by testing against 9 non-*albicans* species (including 3 *Candida* species). It also had a very high sensitivity of 6.2 × 10^−6^ ng/mL, which was 10 times more sensitive than qPCR [149].

### 4.3. Iso-PCR

Iso-PCR is an isothermal technique with two steps: first, a multiplex PCR amplifies one target region per species using multiple primer sets; second, the species-specific PCR amplicon is detected via an isothermal amplification that occurs only if the target sequence is present [150]. The PCR employs a nested design, where inner primers amplify the species-specific sequence from the broader region generated by the outer primers. One study applied iso-PCR for *N. glabratus*, achieving extremely high sensitivity, with detection down to a single DNA copy (Table 9) [150].

### 4.4. RPA

RPA uses target-specific primers bound to recombinase to access double-stranded DNA. A single-stranded DNA-binding protein stabilizes the amplifying strand as *Bacillus subtilis* polymerase then extends the primers, rapidly generating amplicons. RPA occurs isothermally at 30–45 °C, with results detectable in ~20 min via gel electrophoresis or fluorescence detection. RPA advantages include single-temperature amplification and use of only two primers, reducing false positives from primer interactions compared to LAMP [37]. A limitation, like LAMP, is the potential for aerosolized amplicon contamination due to high amplification efficiency [37].

Lateral flow strips (LFS) offer a simple, field-friendly option for species identification with RPA, requiring minimal equipment [35]. Six articles utilized RPA in conjunction with LFS to identify *C. albicans*, *C. tropicalis*, *N. glabratus*, *P. kudriavzevii*, *C. auris*, and *C. parapsilosis* (Table 10) [35,151,152,153,154]. All designed primers targeted the ITS2 gene except for Zhu et al. (2023), who designed primers for the *FKS2* gene [35]. Pre-processing of the samples by DNA extraction was required for all the studies [35].

In Zhu et al. (2023), the RPA-LFS assay demonstrated a LoD of 5.85 × 10^3^ copies/reaction at 95% probability [35]. Specificity for the *C. parapsilosis* assay was tested with 35 common clinical species. Wang et al. (2022a) and (2022b) had a sensitivity of 10 CFU/µL when detecting *N. glabratus* or *C. tropicalis* [152,153]. The *C. albicans* assay achieved the highest sensitivity of 1 CFU/r’x, likely in part due to the addition of a probe which limited primer dimerization and increased sensitivity [151].

### 4.5. RAA-LFS and DO-RAP

RAA combined with lateral flow strips (RAA-LFS) is a similar design compared to RPA (Table 10) [155]. In this case, it was utilized to identify *C. auris* by targeting the ITS region. Similarly, a single-strand displacing recombinase is utilized in combination with single-strand binding proteins and an isothermal DNA polymerase. The NAAT amplicon can be visualized on the LFS within 15 min at a high sensitivity of 1 CFU/reaction and a 100% specificity. Feng et al. (2024) had similar results, with assay sensitivity being 10 copies/μL and 100% specificity [156].

Lyu et al. (2025) describe a dual one-step recombinase-aided PCR (DO-RAP) system combined RAA with M1 protein magnetic beads to first enrich cells from blood cultures before DNA extraction [157]. The mannan-binding lectins (MBL) on the beads bind the cells via mannose and N-acetylglucosamine residues on *Candida* cell surfaces. Unlike using docosane to separate RAA and qPCR steps, DO-RAP integrates both reactions in a single tube, with optimized reagents that allow the RAA reaction to transition seamlessly to the higher-temperature PCR cycles [157].

The target genes for the DO-RAP primers and probes were the 26S ribosomal RNA gene and the *NADH5* mitochondrial gene in *P. kudriavzevii* and *C. parapsilosis*, respectively [157]. This method had ultra-sensitivity at 1 CFU/mL [157]. Wang et al. (2025) detected *C. albicans*, *C. tropicalis*, and *N. glabratus* from clinical blood samples using the M1-mRAP technique [158]. In this study, the RAA and qPCR steps were combined in a single tube, with one RAA primer pair and one TaqMan ^®^ probe (Thermo Fisher Scientific, Foster City, CA, USA) per species. The thermocycler protocol required 15 min at 40 °C for the RAA reaction, followed by enzyme inactivation and qPCR cycling. Although detection took ~3.5 h due to qPCR, the method achieved high sensitivity (1–2 CFU/mL) and allowed multiplexing using differently labeled fluorescent probes [158].

### 4.6. Other Isothermal NAAT Methods: MCDA

With multiple cross displacement amplification (MCDA), an isothermal reaction at 64 °C can be completed in 40 min. However, additional time is necessary to process the sample first with DNA extraction prior to amplification. Zhao et al. (2019) designed and assessed an MCDA assay targeting the ITS2 region using 10 primers (Table 11) [159]. They achieved a very low LoD (200 fg DNA/reaction), surpassing conventional PCR and LAMP in sensitivity (1000 fg DNA/reaction and 500 fg DNA/reaction, respectively). One limitation of MCDA is the risk of contamination from high amplification efficiency. Adding uracil-DNA glycosylase in future iterations could address this issue [159].

### 4.7. Isothermal Techniques with CRISPR Diagnostics

Isothermal techniques like LAMP and RPA can be combined with CRISPR diagnostics to improve assay sensitivity and specificity [37]. While CRISPR-LAMP has been applied to other pathogens, including SARS-CoV-2, it has not yet been used for *Candida* species [160,161,162,163]. One challenge is that optimizing reagent compatibility can take time and resources. Overall, LAMP is a promising approach for identifying clinically relevant *Candida*, with genetic advances that could be combined to further enhance its performance.

Shen et al. (2024) identified *C. albicans* with an affinity molecular assay that combines chitin affinity-magnetic separation with an RPA-CRISPR/Cas12a one-pot design [37]. Prior to the NAAT reaction, fungal cells were captured using magnetic beads covered in chitin-binding proteins that target this major component of the fungal cell wall. DNA was extracted with silicon hydroxyl magnetic beads and then input into RPA with the *CHS1* gene target. The CRISPR gRNA was designed to detect the RPA products and produce a fluorescent signal. Despite the additional steps involved, this platform showed good turnaround time, with the entire process taking about 150 min, and good sensitivity with a threshold of 30 CFU/mL [37].

## 5. Other High-Throughput Methods for *Candida* Species Identification

**Figure 5:**
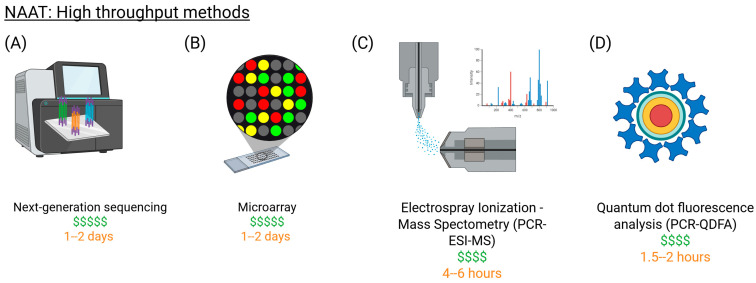
High-throughput methods cited in this review. (**A**) Next-generation sequencing via metagenomic (mNGS) or targeted (tNGS) sequencing. (**B**) Microarray. (**C**) PCR-ESI/MS (electrospray ionization/mass spectrometry). (**D**) PCR-QDFA (quantum dot fluorescence analysis). Created in BioRender (BioRender Inc., Toronto, ON, Canada). Tharmalingam, S. (2026). https://BioRender.com/xscj119 (accessed on 14 May 2026).

Several advanced high-throughput nucleic acid-based technologies have been used for *Candida* species identification. These include next-generation sequencing (NGS) methods, hybridization-based platforms such as microarrays, fluorescence-based detection using quantum dots, and mass spectrometry coupled with electrospray ionization (Figure 5). The defining feature of electrospray ionization mass spectrometry (ESI-MS) and quantum dot fluorescence assay (QDFA) is not the PCR amplification step they share with other methods, but rather the downstream detection strategy that enables multiplexing and species-level resolution.

### 5.1. NGS for Candida Species Identification

Candidiasis-causing fungal species can be detected using targeted NGS (tNGS) or metagenomic (shotgun) NGS (mNGS). Most sequencing studies in this review used mNGS, with *C. albicans* typically the only species identified. The diagnosis of conditions varied, including refractory pneumonia, invasive fungal disease, pulmonary infections, urinary infections, post-lung transplantation infection, and lower respiratory tract infections (Table 12) [164,165,166,167,168,169]. Using NGS to characterize a sample and identify relevant species is a very good option if the timing is not urgent, as this method has very high discriminatory power and high sensitivity. However, NGS methods have longer turnaround times and higher costs, often require outsourcing and specialized bioinformatics expertise, and may produce false positives due to their high sensitivity.

#### 5.1.1. tNGS

To identify *Candida* species with tNGS, conserved fungal barcode regions with high interspecies and low intraspecies variability (most commonly the ITS region or the D1/D2 region of the 28S rRNA) were amplified and aligned to reference databases. Four articles were cited using tNGS [167,170,171,172]. Liu et al. (2024) noted tNGS as a valid option for diagnosing pulmonary infections, considering that it outperformed mNGS with a higher coincidence rate of correct clinical diagnosis (81.4% vs. 40%) [172]. In contrast, Chen et al. (2025) found that when diagnosing respiratory tract fungal infections, mNGS performed slightly better than tNGS in terms of specificity (91% vs. 85%), though they had the same high sensitivity of 95% [171].

#### 5.1.2. mNGS

Alternatively, mNGS sequences the whole genome using DNA samples. Sequencing reads derived from a biological sample are mapped to a corresponding reference genome, allowing simultaneous detection of multiple pathogens such as fungi, bacteria, and viruses. This approach provides highly accurate species identification, can distinguish closely related or cryptic species, and detects organisms at low abundance. A total of 14 articles used mNGS for diagnosing *Candida* fungal infections [164,165,166,167,168,169,171,173,174,175,176,177,178,179].

One drawback in interpreting the diagnostic performance of NGS may be that a smaller sample size or range of possible pathogens results in fewer *Candida* infections identified. For example, Wang et al. (2024) identified four *C. albicans* infections while Zhang et al. (2024) identified only one, so diagnostic performance metrics may be skewed [165,170]. They found that mNGS was much more sensitive than conventional microbiological testing methods (85% vs. 37%), and the turnaround time to diagnosis was reduced [165]. Liu et al. 2024 found 38 *Candida* infections, and overall, NGS had a significantly higher PPV for pathogens compared to the reference method [172]. Lian et al. (2021) had a very high sensitivity for their high-throughput NGS study of 97%; however, specificity (31.3%) was lower and could be improved upon [168]. Li et al. (2025) also tested mNGS, which resulted in a very high sensitivity (approximately 97%) but low specificity at approximately 13% [169]. Meng et al. (2025) compared the Q and PACE mNGS analyses and found that the PACE method was more sensitive for detecting fungal species like *C. albicans* (92%) [177].

### 5.2. Reverse Dot Blot Hybridization and Microarrays

Traditional PCR protocols can be utilized in conjunction with reverse dot blot hybridization (RDBH), a molecular diagnostic technique used to detect specific DNA sequences [180]. Known DNA probes are immobilized onto a membrane, and then the labeled PCR amplicon is added to the reaction to hybridize with complementary probes. A fluorescent signal indicates the presence of that species-specific amplicon. With this method, simultaneous detection of multiple pathogens or genetic mutations is possible, but it cannot identify unknown sequences. A microarray is a high-throughput platform with the same concept as RDBH in which thousands of DNA probes are immobilized on a solid surface (such as a glass slide or chip) [181]. Compared with RDBH, microarrays enable much larger-scale multiplexing but still require predefined target sequences.

Eight articles in this review combined PCR with a reverse dot blot hybridization or microarray setup (Table 13) [180,182,183,184,185,186,187,188]. For example, in Playford et al. (2006), an initial PCR using pan-fungal primers amplified the highly conserved sequences of the ITS region with highly variable portions in between the two primers [180]. Species-specific fluorescent hybridization probes were designed to bind to the amplicon according to one of the thirteen pathogens selected for detection [180].

Xiang et al. (2007) first amplified ITS2 region sequences, and the denatured PCR amplicons were hybridized to the reverse blot [187]. Complementary species-specific oligonucleotides designed to identify *C. albicans*, *C. tropicalis*, *P. kudriavzevii*, *N. glabratus*, *C. parapsilosis*, and *C. dubliniensis* were used, demonstrating success with a LoD of 10 CFU/mL. Zeng et al. (2007) used a nested PCR approach [186]. Their pan-fungal probe bound to any successfully amplified product, and biotin-labeled primers allowed for detection via streptavidin beads on a reverse blot. This method identified two clinical samples that culture failed to speciate. ITS2 probes showed 100% specificity, while ITS1 probes were nearly 100% specific, with minor cross-hybridization between the sibling species *C. haemulonii* and *C. norvegensis*. The sensitivities of the assay were 98% and about 94% for the ITS2- and ITS1-based probes, respectively [186].

Spiess et al. (2007) developed a microarray to identify *C. albicans*, *C. dubliniensis*, *N. glabratus*, *C. lusitaniae*, and *C. tropicalis* [185]. First, multiplex PCR was used to amplify conserved rDNA regions from fungal cells in blood samples, and then the amplicons were hybridized to species-specific capture probes. Detection limits reached as low as ~1 pg per reaction, but this value varied by species, potentially resulting in false negatives due to different hybridization efficiencies. Similarly, Shiang et al. (2007) used pan-fungal ITS2 rDNA PCR with DIG-labeled, species-specific probes on a nylon membrane microarray [188]. This approach could identify up to 77 species among 16 genera, with high reliability. However, this method requires prior culturing, and the cost to create the microarray can be high due to technical complexity.

The study by Lin et al. (2023) utilized PCR and RDBH, resulting in diagnostic performance of 96.7% sensitivity and 100% specificity [182]. The detection limit of 10^3^ CFU/mL may need to be refined if this method is to be clinically useful. Last, the commercial Prove-it™ Sepsis kit (Hyperion Diagnostics/Merlin Diagnostics, Berea, OH, USA) detects candidemia from blood culture samples. Initially developed for bacterial pathogens, it was later expanded to identify seven clinically relevant fungal species using microarray hybridization with fluorescent probes. Most samples were identified at the species level, although a small number were detected only at the pan-yeast taxon level [184].

### 5.3. Quantum Dot Fluorescent Analysis (QDFA)

QDFA is like microarray-based hybridization, but instead of traditional fluorophores, it uses species-specific quantum dot-labeled probes [189]. Following PCR amplification of target fungal DNA, amplicons hybridize to quantum dot probes, allowing for simultaneous detection of multiple *Candida* species based on each distinct fluorescence signal [190]. This enables higher sensitivity and improved multiplexing, as fluorescent signals are more stable and long-lasting. There was 1 article that evaluated QDFA (Table 14) for diagnosing VVC [189]. While sensitivity and specificity were generally good (81–93% and 94–100%, respectively), false positives and negatives were identified when compared with culture plus MALDI-TOF methods [189].

### 5.4. ESI-MS

Electrospray ionization mass spectrometry (ESI-MS) can also be combined with PCR amplification [191]. Amplicons are ionized via electrospray to form gas-phase DNA ions, which are analyzed by mass spectrometry and matched to a reference database using mass-to-charge ratios [192]. Although PCR amplification is required to generate detectable nucleic acid material, the downstream mass spectrometric analysis provides high throughput and discriminatory power, which enables multiplexed and culture-independent identification of *Candida* species. A key limitation is that only species included in the database can be identified.

Four articles used PCR-ESI/MS (Table 14) [193,194,195,196]. Gu et al. (2012) assessed rep-PCR and PCR/ESI-MS using ITS sequencing as the gold standard, correctly identifying 51 of 61 samples in both methods [193]. Accuracy reached ~98% when limited to species present in the reference database, though clinical feasibility was not addressed. Similarly, Shin et al. (2013) applied PCR/ESI-MS in the PLEX-ID assay (Abbott Diagnostics, Des Plaines, IL, USA) to detect *Candida* in bronchoalveolar lavage samples [194], while Strålin et al. (2020) extended its use to blood samples, highlighting its potential for broad pathogen detection in sepsis [195]. This study’s assay had true-positive and true-negative results of 99% and 97%, respectively. Specificity was found to be above 94% for each species.

## 6. Key Findings

A major finding of this review is that multiplexing should be prioritized whenever feasible, as it reduces the labor, time, and resources required to detect and differentiate multiple *Candida* species within a single assay. High analytical sensitivity is particularly important for identifying polymicrobial infections, where one or more species may be present at low abundance, and is also advantageous when only limited patient sample volumes are available. In parallel, short turnaround times improve the potential for near-patient or point-of-care implementation, which is critical for timely clinical decision-making. Although highly sensitive and specific platforms such as next-generation sequencing offer broad and powerful detection capabilities, they may be more suitable in settings where immediate turnaround is less critical. Importantly, specificity remains paramount for the clinical utility of any *Candida* species identification assay. Substantial interspecies differences exist, enabling assays that can accurately distinguish target species. However, cross-reactivity remains possible, and careful primer design is essential. Overall, this review features a range of diverse NAAT platforms for *Candida* species identification. Key performance characteristics and assays with high diagnostic potential are emphasized to help guide future assay development.

To highlight the most promising diagnostic options, top-performing studies for the identification of clinically relevant *Candida* species were summarized by time to result, limit of detection, sensitivity, and specificity. These assays show strong diagnostic potential and suggest which NAAT platforms may best support rapid, accurate, species-specific detection in clinical settings.

*C. albicans:*

○ Schabereiter-Gurtner et al. 2007 melting curve analysis, time of 20 min, LoD 0.001 CFU/µL, 100% specificity [40]
○ Jin et al. 2023 LAMP, time of 24.5 min, LoD of 0.001 CFU/µL, 100% specificity [144]
○ Wang et al. 2021 RPA, time of 20 min, LoD of 0.001 CFU/µL, 100% specificity [151]

*N. glabratus*:

○ Wang et al. 2025, RPA, time of 18.25 min, LoD of 0.001 CFU/µL, 100% specificity [158]
○ Jin et al. 2023 LAMP, time of 24.5 min, LoD of 0.001 CFU/µL, 100% specificity [144]
○ Klingspor et al. 2006, melting curve analysis, time of 23 min, LoD of 0.002 CFU/µL, 83.5% sensitivity, 100% specificity [121]

*P. kudriavzevii*:

○ Jin et al. 2023 LAMP, time of 24.5 min, LoD of 0.001 CFU/µL, 100% specificity [144]
○ Klingspor et al. 2006, melting curve analysis, time of 23 min, LoD of 0.002 CFU/µL, 83.5% sensitivity, 100% specificity [121]
○ Zhao et al. 2022, RPA-LFS, time of 20 min, LoD of 0.2 CFU/µL, 100% specificity [154]

*C. auris*:

○ Zhang et al. 2023 RPA, time of 15 min, LoD of 0.001 CFU/µL, 100% specificity [155]
○ Yamamoto et al. 2024, LAMP, time of 60 min, LoD of 0.008 CFU/µL, 86% sensitivity, 100% specificity [148]

*C. tropicalis:*

○ Jin et al. 2023 LAMP, time of 45 min, LoD of 0.002 CFU/µL, 100% specificity [144]
○ Klingspor et al. 2006, melting curve analysis, time of 23 min, LoD of 0.002 CFU/µL, 100% specificity [121]
○ Guo et al. 2016, PCR and probes, time of ~60 min, LoD of 0.01 CFU/µL, 100% sensitivity, 98.4% specificity [76]

*C. parapsilosis*:

○ Jin et al. 2023 LAMP, time of 24.5 min, LoD of 0.001 CFU/µL, 100% specificity [144]
○ Klingspor et al. 2006, melting curve analysis, time of 23 min, LoD of 0.002 CFU/µL, 83.5% sensitivity, 100% specificity [121]

## 7. Future Directions and Conclusions

The findings of this scoping review demonstrate the expanding clinical utility of NAATs for the identification of *Candida* infections. Substantial variability in sensitivity, specificity, and overall diagnostic performance across studies and clinical contexts was also observed. The included studies spanned diverse geographic regions with differences in diagnostic strategies that are likely influenced by resource availability, assay design, and specimen type. Most NAAT workflows also require nucleic acid extraction and lysis steps, which may limit implementation. Furthermore, optimization of reagents and careful primer design are necessary to maximize both sensitivity and specificity.

Molecular targets such as ITS1, ITS2, and 18S/28S rDNA regions were identified as common biomarkers for *Candida* identification. RT-PCR and LAMP-LFB demonstrated 100% sensitivity and specificity, making them worth further exploration as reliable diagnostic methods. Other methods like multiplex qPCR showed highly variable performance metrics. NAAT methods with low LoDs, such as LAMP-LFB (1 fg/μL), show promise for enhanced identification in low-fungal-load infections. Diagnostic performance was evaluated across broad clinical contexts, including high-risk groups such as ICU patients and individuals with hematological malignancies, as well as cases of vaginal candidiasis, peritonitis, and systemic candidiasis. Variable test performance may reflect the diagnostic complexity of these conditions.

Despite promising findings, the limitations of this review include variability in diagnostic performance, while methodological differences (e.g., NAAT type, reference method, sample collection, and gene target choice) hamper direct comparisons across studies. Future research should prioritize standardization of methods and targets and conduct multicenter studies to validate findings across diverse populations. Of further importance is the evaluation of the cost-effectiveness of lower-cost assays like LAMP-LFB for application in resource-limited settings. Some clinical and hospital settings do not have access to fully equipped microbiological laboratories with the necessary equipment and technicians. Feasibility ultimately needs to be considered when selecting a NAAT for *Candida* species identification. The development of kits that can easily be stored at room temperature, with lyophilized reagents and simple lateral flow transfer to visualize results, could provide more accessible options for such healthcare environments.

Another limitation of this review is the restriction to studies using clinical samples, which excluded investigations relying solely on spiked or simulated specimens despite reporting diagnostic performance. For example, Olchawa et al. (2023) evaluated NAAT-based detection using non-clinical samples and was therefore not included [197].

An important clinical consideration is the commensal nature of *C. albicans* and others, which complicates interpretation of molecular detection. Baseline fungal colonization levels are not well defined, and the threshold distinguishing commensal presence from pathogenic infection remains unclear. For instance, Czajka et al. (2025) reported that several clinical samples that originated from symptomatic individuals were instead classified as normal flora [4]. This underscores the need to interpret NAAT results in the context of clinical presentation, patient risk factors, and complementary diagnostic data. Future studies should aim to establish clinically relevant fungal load thresholds to differentiate colonization from infection and inform sensitivity thresholds for diagnostics.

Highly sensitive NAATs may also improve detection of polymicrobial infections and enable early identification of intrinsically resistant species such as *N. glabratus* and *P. kudriavzevii*. Early detection of these low-abundance resistant species could guide more appropriate antifungal selection to treat the entire infection. Additionally, ultra-sensitive detection of *C. auris* is critical for infection control, given its multidrug resistance and non-commensal nature. Overall, continued advancements in NAAT design, combined with improved clinical interpretation frameworks, will be essential to fully realize the potential of *Candida* species identification in *Candida* diagnostics.

## Figures and Tables

**Table 1:** Summary of diagnostic factors for papers using primarily standard PCR techniques.

Study ID	1st Author Name	Year	NAAT Type	Condition Diagnosed	Species	Gene Target	Sensitivity	Specificity
1	Landlinger	2009a	PCR	Fungal infection	*Candida* spp.	ITS2 rDNA	1 fg DNA	?
2	Salman	2010	PCR	Pediatric fungal endophthalmitis	*C. albicans*	18S rDNA	100%	100%
3	Martínez	2010	PCR	Blood cultures	*C. albicans* *N. glabratus*	RPS0 gene intron	1 pg DNA 10^3^ cells	100%
4	Pilar Vercher	2011	PCR	Blood cultures	*C. parapsilosis C. orthopsilosis* *C. metapsilosis*	RPS0 gene intron	?	100% (only tested one other species)
5	Kühn	2011	PCR	Endocarditis/blood cultures	*C. albicans* *N. glabratus*	18S-28S rDNA	85% PPV 73.9%	40% NPV 57.1%
6	Ahmad	2012	PCR	Clinical yeast isolates	*C. dubliniensis* *C. albicans*	ITS rDNA	97–100%	100%
7	Cartwright	2013	PCR	VVC	*C. albicans* *N. glabratus*	ITS2-Alb ITS1-Glab	97.7% PPV 91.3%	93.2% NPV 76.6%
8	Xafranski	2013	PCR	Blood cultures	5 *Candida* spp.	ITS1, 5.8s, ITS2, ITS4 rDNA	10 pg/10 CFU/mL—Alb	95–Alb
9	Bineshian	2015	PCR	HIV patients, VVC	*C. albicans* *C. parapsilosis* *N. glabratus*	MP65 gene	50 yeast cells/r’x	100%
10	Obručo-vá	2016	PCR-CE	Clinical yeast isolates	*Candida* spp.	ITS2 rDNA	100%	100%
11	Kordalewska	2017	PCR	Clinical yeast isolates	*C. auris*	5.8S, ITS2, 28S rDNA	100% 10 CFU/mL	100%
12	Ruiz-Gaitán	2018	PCR	Blood cultures + other	*C. auris*	GPI protein genes	10 pg of DNA	100%
13	Garcia-Salazar	2022	PCR	Clinical yeast isolates	8 *Candida* spp.	ITS rDNA	73.9% 10^3^ cells/mL PPV: 94.4%	96.30% NPV: 81.2%
14	Komorowski	2024	PCR	Clinical yeast isolates	*C. auris*	16S rDNA	82.1% 10 CFU/mL	100%
15	Gentile	2013	LATE-PCR	Clinical yeast isolates	5 *Candida* spp.	?	100%	?
16	Mohammadi	2015	DGGE, TTGE PCR	Vaginal swabs	5 *Candida* spp.	ITS2 rDNA	20 ng DNA	100% NPV 96.55%
17	Srichaiyapol	2025	short-PCR-LFS	Blood culture samples	*C. albicans*	ITS2 rDNA	2 CFU/mL PPV 100%	

? Symbol represents missing data.

**Table 2:** Summary of nested and semi-nested PCR techniques.

Study ID	1st Author Name	Year	NAAT Type	Condition Diagnosed	Species	Gene Target	Sensitivity	Specificity
18	Alam	2007	snPCR	blood culture samples	*C. albicans* *C. tropicalis* *C. parapsilosis*	5.8S-28S rDNA	88%	100%
19	Landlinger	2009b	snPCR- Luminex^®^	invasive fungal infection	5 *Candida* spp.	ITS2 rDNA	10–100 fg DNA 0.3–3 pathogens	?
20	Ebihara	2009	Nested PCR	onychomycosis	*C. albicans* *C. tropicalis*	ITS1 rDNA	?	100%
21	Çerikçioǧlu,	2010	snPCR	blood cultures	*C. parapsilosis* *C. albicans*	ITS2 rDNA	100%	97%
22	Gosiewski	2014	Nested qPCR	blood infections	*C. albicans*	16S, 18S rDNA	10 CFU/mL	100%
23	Taira	2014	Nested PCR	blood cultures	7 *Candida* spp.	ITS1-ITS4 rDNA	4 genomes/mL	100%
24	Badiee	2016	Nested PCR	invasive fungal infection	*C. albicans* *C. tropicalis*	ITS1-ITS4 rDNA	84.6%, PPV 91.7%	88.8% NPV 80%
25	Caméléna	2023	Nested-PCR (FilmArray^®^ BCID2)	bloodstream infections	6 *Candida* spp.	*bla*CTX-M, major carbapenemase genes	67–100%	100%
26	Tirard-Collet	2025	DendrisKIT^®^ DP	skin fungal infection	*C. albicans*	ITS rDNA	83.90%	88.90%

? Symbol represents missing data.

**Table 3:** PCR techniques with the use of Restriction Fragment Length Polymorphism.

Study ID	1st Author Name	Year	NAAT Type	Condition Diagnosed	Species	Gene Target	Sensitivity	Specificity
27	Szemiako	2017	PCR-RFLP	yeast clinical samples	5 *Candida* spp.	homocitrate synthase gene	?	100% ?
28	Sadrossadati	2018	PCR-RFLP	candidemia	9 *Candida* spp.	ITS1-5.8S rDNA ITS2	96.6% ?	?
29	Marcos-Arias	2020	PCR-RFLP	blood cultures	*C. parapsilosis*	SADH and FKS1 genes	97% and 99% respectively	100% and 78% respectively
30	Jabrodini	2024	PCR-RFLP	Invasive fungal disease	*C. albicans* *C. parapsilosis* *C. tropicalis*	ITS1-5.8S-ITS2 rDNA	88.50%	100%

? Symbol represents missing data.

**Table 4:** Summary of diagnostic factors for papers using primarily qPCR/Rt-PCR with fluorescent dye techniques.

Study ID	1st Author Name	Year	NAAT Type	Condition Diagnosed	Species	Gene Target	Sensitivity	Specificity
31	Shuping	2023	SYBR PrimeScript^TM^ RT-PCR	fungal infection	*C. auris*	?	44% PPV 97%	99% NPV 79%
32	Felix	2023	RT-PCR	blood samples	5 *Candida* spp.	ITS rDNA	10 copies/assay	100%

? Symbol represents missing data.

**Table 5:** Summary of PCR techniques with the use of DNA probes.

Study ID	1st Author Name	Year	NAAT Type	Condition Diagnosed	Species	Gene Target	Sensitivity	Specificity
33	McMullan	2008	TaqMan^TM^ RT-PCR	Axilla groin swabs	6 *Candida* spp.	18S-ITS2 rDNA	90.9% PPV 100%	100% NPV 99.8%
34	Badiee	2009	PCR-ELISA	Blood culture samples	*C. tropicalis* *P. kudriavzevii* *C. albicans*	18S-28S rDNA	84.60% PPV 75.3%	92.70% NPV 95.8%
35	Badiee	2011	TaqMan^TM^ RT-PCR	Nosocomial fungal infection	*Candida* spp.	rDNA	86.60%	82%
36	Guo	2016	Probe RT-PCR	Blood culture samples	5 *Candida* spp.	rDNA	100%	98.40%
37	Leach	2018	TaqMan^TM^ Probe RT-PCR	Surveillance samples	*C. auris*	ITS2 rDNA	89% 1 CFU/r’x PPV 94%	99% NPV 98%
38	Martínez-Murcia	2018	GPS^TM^ MONODOSE CanAur dtec qPCR	Clinical yeast samples	*C. auris*	Proprietary	5–10 DNA copies/r’x	100%
39	Leach	2019	BD Max^TM^ assay-probe RT-PCR	Surveillance samples	*C. auris*	ITS rDNA	96% 1 CFU/r’x PPV 92%	92% NPV 97%
40	Ahmad	2019	TaqMan^TM^ Probe RT-PCR	Dermal swabs	*C. auris*	ITS2 rDNA	93.6% 1 CFU/10 μL	97.20%
41	Sattler	2021	qPCR AurisID^®^ and Fungiplex^®^		*C. auris*	28S rDNA	1 genome copy/r’x 9 copies/rx	AurisID gave some false positives
42	Mulet Bayona	2021	RT-PCR AurisID^®^	Pharyngeal or axillary-rectal swab	*C. auris*	28S rDNA	96.6% 500 CFU/mL PPV 100%	100% NPV 98.2%
43	Ibrahim	2021	TaqMan^TM^ Probe RT-PCR	Clinical yeast samples	*C. auris*	GPI protein gene	13 CFU/r/x	100%
44	Zheng	2023	TaqMan^TM^ Probe PCR	Sepsis	*C. albicans* *C. tropicalis*	?	1–10 CFU/mL	Lacking
45	Rosa	2023	DiaSorin (Simplexa^®^) PCR	Axilla groin swabs	*C. auris*	ITS2 rDNA	600 CFU/mL	100%
46	Ramirez	2023	DiaSorin (Simplexa^®^) PCR	Axilla groin swabs	*C. auris*	ITS2 rDNA	100% 266 CFU/mL PPV 100%	100% NPV 100%
47	Leonhard	2024	TaqMan^TM^ Probe RT-PCR	Skin swabs	*C. auris*	ITS rDNA	4 cells per PCR	100% (22 other Candida)
48	Banik	2024	Molecular beacon probe RT-PCR-*CaurisSurV*	Skin swabs	*C. auris*	ITS2 rDNA	97.5% 10.5–14.8 CFU/mL	100%
49	Franco	2024	DiaSorin (Simplexa^®^) PCR	Axilla, inguinal, or other swabs	*C. auris*	ITS2 rDNA	100% 1–2 CFU/rx PPV 100%	100% NPV 100%
50	Nhan	2025	TaqMan^TM^ probe RT-PCR	Nasal swabs	*C. auris*	ITS2 rDNA	1.95 Log CFU/mL PPV 100%	100% NPV 100%
51	Dehesa-Garcia	2025	VIASURE^®^ probe RT-PCR	Axilla, inguinal swabs + others	*C. auris*	ITS2	98%	100%

? Symbol represents missing data.

**Table 6:** Summary of multiplex PCR and multiplex RT-PCR techniques.

Study ID	1st Author Name	Year	NAAT Type	Condition Diagnosed	Species	Gene Target	Sensitivity	Specificity
52	Liguori	2007	Multiplex PCR	Oral rinse samples	*Candida* spp.	ITS	10 CFU/mL	70.30%
53	Carvalho	2007	Multiplex PCR	Clinical yeast samples	8 *Candida* spp.	ITS1 and ITS2	2 cells per ml	100%
54	Innings	2007	Multiplex TaqMan^TM^ Probe RT-PCR	Blood samples	7 *Candida* spp.	RNase P	1–10 genome copies	100% ?
55	Sugita	2012	Multiplex TaqMan^TM^ Probe RT-PCR	Fungal endophthalmitis	*C. albicans*	18S rDNA	10^1 per PCR	100%
56	Balada-Llasat	2012	Multiplex PCR-Luminex xTAG Fungal Assay	Blood cultures	7 *Candida* spp.	HWP1, RNase P	100%—PPV 99%	99%—NPV 100%
57	Sitnik	2014	Septi-fast	Sepsis	*C. albicans* *N. glabratus* *C. tropicalis*	ITS	81.3% PPV 100%	100%—NPV 93.5%
58	Monstein	2014	PCR-CE	Clinical yeast isolates	6 *Candida* spp.	ITS2		
59	Fortún	2014	Beacon probe multiplex RT-PCR	Invasive candidiasis	6 *Candida* spp.	ITS	96.3% PPV 92.8%	97.3% NPV 98.7%
60	Schmitt	2014	Multiplex PCR-Luminex^®^	STI	3 *Candida*	?	97%	99%
61	Vahidnia	2015	Multiplex PCR	Toenail fungal infection	*C. albicans* *C. parapsilosis*	Ker1, NADH5	100% PPV 98.53%	99.32% NPV 100%
62	Fukumoto	2015	Multiplex TaqMan^TM^ RT-PCR	Lung tissue samples	*C. albicans*	5.8S rDNA	10 amplicon copies/r’x	>95%
63	Mutschlechner	2016	Multiplex RT-PCR	Mammary candidiasis	7 *Candida* spp.	ITS2	67.4%	41.9%,
64	Elges	2017	Multiplex RT-PCR Septifast	Sepsis	*C. albicans* *P. kudriavzevii* *C. parapsilosis*	ITS	39.1% PPV 60%	99.3% NPV 93%
65	Sampath	2017	Multiplex PCR	Oral rinse samples	*C. albicans*, *C. tropicalis*, *C. parapsilosis* *N. glabratus*	ITS1/ITS2	94.4% PPV 80%	39.3% NPV 72%
66	Nakano	2017	Strip-PCR	Ocular infectious disease	*Candida* spp. *P. kudriavzevii* *N. glabratus*	28S rDNA	50 DNA copies/r’x	100% ?
67	Masha	2018	TAC probe qPCR	Fungal infection	*C. albicans*	?	85.70%	?
68	Arastehfar	2018	Multiplex end-point PCR	Clinical yeast isolates	*C. auris*, *C. haemulonii*, *C. duobushaemulonii*, *C. pseudohaemulonii*	26s rDNA	?	100%
69	Hayette	2019	Multiplex RT-PCR Derma-Genius^®^	Onychomycosis	*C. albicans*	ITS	80%	74.40%
70	Liotti	2019	Multiplex RT-PCR MicrobScan	Blood culture	*C. albicans* *N. glabratus* *P. kudriavzevii* *C. parapsilosis*	ITS1, 5.8S ITS2 rDNA	86.4% PPV 91.1%	97% NPV 95.3%
71	Fuchs	2019	Multiplex RT-PCR Fungiplex^®^	Blood culture	*P. kudriavzevii* *N. glabratus* *Candida* spp.	Proprietary	100% PPV 63%	94.1% NPV 100%
72	Arastehfar	2019	Multiplex PCR	Clinical yeast samples	*Candida* spp.	ITS ?	Implied 100%	100% ?
73	Carvalho-Pereira	2020	Multiplex PCR	Invasive fungal infection	5 *Candida* spp.	?	89% 1–10 pg DNA	100%
74	Ndiaye	2021	Multiplex RT-PCR Derma Genius^®^	Onychomycosis	*C. albicans*	ITS	89.3% PPV 73.3%	75.3% NPV 90.2%
75	Aboutalebian	2021	Multiplex PCR	Otitis externa	*C. albicans*, *C. parapsilosis*, *C. auris*	ITS	?	100 ?
76	Jahn	2022	Multiplex RT-PCR, Septi-fast	Sepsis	5 *Candida* spp.	ITS rDNA	30–100 CFU/mL	?
77	Aboutalebian	2022	Multiplex PCR, YEAST PLEX	Clinical yeast samples	*Candida* spp.	ITS or IGS	?	100%
78	Choi	2022	AccuPower^®^ STI4CPlex RT- PCR	Clinical yeast samples	*C. albicans*	?	147.9 copies/mL	?
79	Li	2023a	Multiplex PCR + biochip	Sepsis	*C. albicans*	ITS rDNA	92.9% 20 CFU/r’x PPV 72.2%	93.2% NPV 98.6%
80	Bui	2023	Multiplex RT-PCR-probes	STI	*C. albicans*		100%—8–58 copies	100%
81	Amor	2024	Multiplex RT-PCR-probe-Vircell Vaginal Panel	Vaginitis	*C. albicans* *P. kudriavzevii* *N. glabratus*	?	96% PPV 95.3%	98.4% NPV 98.7%
82	Garcia-Salazar	2025	Cand-PCR	VVC	8 *Candida* spp.	?	65% PPV 100%	100% NPV 91%
83	Camp	2024	Septifast T2MR Candida panel	Sepsis	5 *Candida* spp.	ITS rDNA	68.2% 30 CFU/mL	99%

? Symbol represents missing data.

**Table 7:** Summary of PCR techniques used with high-resolution melting analysis (HRMA).

Study ID	1st Author Name	Year	NAAT Type	Condition Diagnosed	Species	Gene Target	Sensitivity	Specificity
85	Bu	2005	RT-PCR-HRMA	febrile neutropenic patients	*C. albicans*, *P. kudriavzevii*, *C. tropicalis*	ITS1, ITS2 and SSU rDNA	0. 1 pg DNA	100%
86	Klingspor	2006	RT-PCR-HRMA	clinical yeast samples	10 *Candida* spp.	18S rDNA	2 CFU/mL	100%
87	Schabereiter-Gurtner	2007	PCR-HRMA	clinical yeast samples	6 *Candida* spp.	ITS2 rDNA	1 CFU/rx	100%
88	Lau	2010	Multiplex PCR-HRMA	candidemia	10 *Candida* spp.	ITS1, ITS2, EF1A, Beta-tubulin	75% PPV 95%	97% NPV 85%
89	Fricke	2010	RT-PCR-HRMA	clinical yeast samples	5 *Candida* spp.	ITS rDNA	2 genome equivalents/assay	100%
90	Foongladda	2014	Multiplex RT-PCR-HRMA	clinical yeast samples	*C. albicans* *C. tropicalis* *N. glabratus* *C. parapsilosis*	18S rDNA	1–3 CFU/r’x	100%
91	Ninghui	2015	PCR-HRMA	clinical yeast samples	6 *Candida* spp.	ITS rDNA	1 fg/µ	100%
92	Nemcova	2015	RT-PCR-HRMA	clinical yeast samples	25 *Candida* spp.	ITS2 rDNA	50 ng-1 pg	?
93	Ashrafi	2015	FRET probe RT-PCR-HRMA	invasive candidiasis-blood	*P. kudriavzevii* *C. tropicalis* *C. parapsilosis* *C. albicans*	18S rDNA	100 CFU/mL /10 fg DNA	100%
94	Zhang	2016	RT-PCR-HRMA	clinical yeast samples	8 *Candida* spp.	ITS1, ITS2, 28S rDNA	10 fg DNA	0 or n.d. ?
95	Fidler	2018	CanTub-simplex PCR-HRMA	clinical yeast samples	7 *Candida* spp.	ITS, 18S, 28S rDNA	0.2–2 genomic equivalent	
96	Nawar	2020	HRMA-PCR	Blood cultures	*C. tropicalis* *N. glabratus* *P. kudriavzevii*	ITS2 rDNA	24.6% PPV 100%	100% NPV 50%
97	Wen	2020	RT-PCR-MCA	blood cultures	6 *Candida* spp.	ITS	21–73 CFU/mL	100%
98	Walchak	2020	PCR-HRMA	surveillance samples	*C. auris*	ITS2	≤100 CFU/r’x	100%
99	Alvarado	2021	Multiplex-PCR-HRMA	clinical yeast samples	*C. auris*	GPI-protein	5 CFU/r’x	100% ?
100	Mustafayev	2024	PCR-HRMA	candidemia	*C. albicans* *C. dubliniensis*	ITS, D1/D2	75% 10 CFU/mL PPV 37.5%	93% NPV 98.5%
101	Trovato	2025	OLM CandID RT-PCR	Serum samples	6 *Candida* spp.	?	83.3% PPV 87.5%	94.3% NPV 91.7%

? Symbol represents missing data.

**Table 8:** Summary of droplet digital PCR techniques.

Article	1st Author Name	Year	NAAT Type	Condition Diagnosed	Species	Gene Target	Sensitivity	Specificity
102	Schell	2012	dd RT-PCR	blood culture samples	*C. albicans*	ITS1	5–10 cells	94%
103	Chen	2021	ddPCR	blood culture samples	*C. albicans*	ITS ?	4.5 DNA copies/r’x	high

? Symbol represents missing data.

**Table 9:** Summary of Isothermal Techniques using LAMP (Loop-mediated Isothermal amplification).

Study ID	1st Author Name	Year	NAAT Type	Condition Diagnosed	Species	Gene Target	Sensitivity	Specificity
104	Søe	2013	IsoPCR	clinical yeast samples	*N. glabratus*	26S rDNA	1 copy	?
105	Noguchi	2017	LAMP	oral candidiasis	*C. albicans*	ITS2	1 pg	?
106	Fallahi	2020	LAMP	clinical yeast samples	*C. albicans*	ITS2	10 fg	100%
107	Wang	2021a	LAMP-LFB	sputum samples	*C. tropicalis*	ITS2	10 fg DNA	100%
108	Wang	2022a	LAMP-LFB	blood, urine and sputum samples	*C. albicans*	ITS2	1 fg DNA	100%
109	Ganguli	2022	LAMP	Blood infection	*C. albicans*	?	100% 1.2 (CFU)/mL	100%
110	Xu	2022a	LAMP microfluidics	clinical yeast samples	*C. albicans*	ITS	1.11 CFU/mL	
111	Li	2023b	LAMP	VVC	*C. albicans* *N. glabratus* *C. tropicalis*	ITS	90.91% PPV 100%	100% NPV 93.4%
112	Jin	2023	LAMP	VVC	*C. albicans* *C. tropicalis* *C. parapsilosis* *N. glabratus*	ITS	100% <2 CFU/reaction	100%
113	Yahaya	2024	LAMP	clinical yeast samples	*N. glabratus*	ITS2	2.25 copies/µL	100%
114	Berlau	2024	LAMP	blood cultures	5 *Candida* spp.	cox1, cox3, nad5, ITS	93.9% PPV 98.2%	99.3% NPV 97.8%
115	Hernández-Felices	2024	LAMP Eazyplex^®^	clinical samples	*C. auris*	-	91.8% PPV 98.2%	98.8% NPV 94.5%
116	Wang	2024a	ERT-LAMP	Sputum samples	*C. albicans*	ITS2	500 ag/µL	100%
117	Yamamoto	2024	LAMPAuris	Skin swabs	*C. auris*	PFOR	66–86% 8 cells/r’x	97–100%
118	Zhang	2025	CDA	Clinical isolates	*C. albicans*	ITS2	100% 6.2 × 10^6^ ng/mL DNA	100%

? Symbol represents missing data.

**Table 10:** Summary of Recombinase-Aided Amplification (RAA) techniques.

Study ID	1st Author Name	Year	NAAT Type	Condition Diagnosed	Species	Gene Target	Sensitivity	Specificity
119	Wang	2021b	RPA-LFS + probe	Clinical yeast samples	*C. albicans*	ITS2	100% 1 CFU/r’x	100%
120	Wang	2022b	RPA-LFS	Clinical yeast samples	*C. tropicalis*	ITS2	9.94 CFU/µL	100%
121	Zhao	2022	RPA-LFS	Sputum samples	*P. kudriavzevii*	ITS2	10 CFU/50 µL/r’x	100%
122	Wang	2022c	RPA-LFS	Clinical yeast samples	*N. glabratus*	ITS2	10 CFU/µL	100%
123	Zhang	2023	RAA-LFS	Simulated blood, nasal and urine samples	*C. auris*	ITS	1 CFU or 10 fg/rx	100%
124	Zhu	2023	RPA-LFS	Sputum samples	*C. parapsilosis*	FKS2	5.85 × 10^3^ copies/r’x	100%
125	Shen	2024	RPA-CRISPR	Clinical yeast samples	*C. albicans*	Chitin binding protein ?	93.93% 30 CFU/mL PPV 100%	100% NPV 95.5%
126	Feng	2024	RAA	Fungal infection	*C. auris*	18S-28S rDNA	10 copies/µL 10 CFU/mL	100%
127	Lyu	2025	DO-RAP	Blood clinical samples	*P. kudriavzevii* *C. parapsilosis*	26rRNA and NADH5	10^−7^ ng/μL	100%

? Symbol represents missing data.

**Table 11:** Summary of Other Isothermal NAAT Methods.

Study ID	1st Author Name	Year	NAAT Type	Condition Diagnosed	Species	Gene Target	Sensitivity	Specificity
128	Zhao	2019	MCDA-LFB	Clinical yeast samples	*C. albicans*	ITS2	100% 200 fg	100%

**Table 12:** Summary of next-generation sequencing techniques for the identification of *Candida* species.

Study ID	1st Author Name	Year	NAAT Type	Condition Diagnosed	Species	Sensitivity	Specificity
129	Lian	2021	HT-mNGS	Pulmonary infections	*Candida* spp.	97.14% PPV 75.56%	31.25% NPV 83.3%
130	Xu	2022b	mNGS	Pulmonary infections	*Candida* spp.	100%	64.90%
131	Lu	2023	mNGS	Bronchoalveolar lavage fluid (BALF) samples	*C. albicans* *N. glabratus* *C. parapsilosis*	84.81% pathogen detection rate	high
132	Chang	2023	mNGS	Sputum samples	*C. albicans*	**85%**	
133	Wang	2024d	mNGS	Refractory pneumonia	*C. albicans*	95.45% PPV 80.77%	37.5% NPV 75%
134	Zhang	2024	mNGS	Invasive fungal disease	*C. albicans* *C. tropicalis*	84.78%	-
135	Liu	2024	tNGS-multiplex PCR	BALF and sputum samples	*C. albicans* *N. glabratus*	50.80%	18%
136	Wang	2024e	tNGS-Fungifast^®^	Blood samples	*C. albicans*	high	high
137	Li	2024	mNGS	Clinical yeast samples	*C. parapsilosis* *C. tropicalis*	high	low
138	Zhou	2024	mNGS	Severe pneumonia		98.1% PPV 91.4%	16.7% NPV 50.0
139	Wei	2024	mNGS	Acute infections/sepsis	*C. albicans*	92.9%	75.90%
140	Chen	2025	mNGS and tNGS	Sputum samples	*C. albicans*	95.08% PPV: 99.0%, 95.0%	90.74% and 85.19%
141	Meng	2025	mNGS, Q and PACE detection	BALF samples	*C. albicans*	83% (Q method)	70% (Q method)
142	Xing	2025	mNGS	Severe pneumonia	*C. albicans* *N. glabratus* *C. parapsilosis*	69.39% PPV 50.75%	60.24% NPV 76.92%
143	Li	2025	mNGS	Lower respiratory infections	*C. albicans*	97.01%	13.04%
144	Zheng	2025	mNGS	Pneumonia	*C. albicans*	79.05% PPV 98.32	90% NPV 36.73
145	Chang	2025	tNGS	Urinary tract infection	*C. tropicalis* *N. glabrata*	PPV 80%	53.1% NPV 100%

**Table 13:** Summary of PCR combined with Reverse dot blot hybridization and Microarray techniques.

Study ID	1st Author Name	Year	NAAT Type	Condition Diagnosed	Species	Gene Target	Sensitivity	Specificity
146	Playford	2006	PCR + RLB assay	Clinical yeast samples	16 *Candida* spp.	5.8S, ITS2, 28S rDNA	100.5 cells/mL	?
147	Shiang	2007	PCR-microarray	Clinical yeast samples	*Candida* spp.	ITS1, ITS2	97%	100%
148	Xiang	2007	PCR and hybridization probes	Clinical yeast samples	6 *Candida* spp.	ITS2	10 CFU/mL	100%
149	Zeng	2007	PCR-RLB	Clinical yeast samples	16 *Candida* spp.	ITS1, ITS2	100%	very high
150	Spiess	2007	PCR + DNA microarray	Clinical yeast samples	5 *Candida* spp.	ITS	1 pg	100% ?
151	Aittakorpi	2012	Prove-it Sepsis-microarray PCR	Sepsis	6 *Candida* spp.	?	99%	98%
152	Han	2014	PCR-microarray	Onychomycosis	*C. albicans*	ITS	100% PPV 90.5%	84.6% NPV 100%
153	Lin	2023	PCR-RDBH	Blood culture	8 *Candida* spp.	ITS	96.7% 10^3 CFU/mL	100%

? Symbol represents missing data.

**Table 14:** Summary of other high-throughput PCR methods.

Study ID	1st Author Name	Year	NAAT Type	Condition Diagnosed	Species	Gene Target	Sensitivity	Specificity
154	Gu	2012	rep-PCR PCR/ESI-MS	Clinical yeast samples	13 *Candida* spp.	?	98.1% accuracy 0.5–5 pg/sample	
155	Shin	2013	PCR/ESI-MS	BALF samples	*Candida* spp.	LSU	87.2% (genus level)	19.4% (dual positive rate)
156	Strålin	2020	PCR/ESI-MS	Blood cultures	*C. albicans*	?	83%	94%
157	Bacconi	2014	PCR/ESI-MS	Blood cultures	*N. glabratus*	?	83%	94%
158	Fan	2023	PCR-QDFA	VVC	*C. albicans* *N. glabratus* *C. tropicalis* *P. kudriavzevii*	?	88.54% PPV 92%	91.59% NPV 87.39%

? Symbol represents missing data.

## Data Availability

No new datasets were generated for this study. The data supporting the findings of this systematic review are available within the published article and its Supplementary Materials. Additional screening and data extraction records are available from the corresponding author upon reasonable request.

## Supplementary Materials

The following supporting information can be downloaded at: https://www.mdpi.com/article/10.3390/pathogens15070753/s1, S1: PRISMA2020 Checklist [198]; S2: Traffic light plot for risk of bias assessment in PCR methods; S3: Traffic light plot for risk of bias assessment in isothermal methods; S4: Traffic light plot for risk of bias assessment in high throughput methods.
