# Supplementary figures and images for: Nucleic Acid Amplification Tests for Candida Species Identification: A Systematic Review of Diagnostic Performance
Source: Pathogens. 2026 Jul 17;15(7):753. doi: 10.3390/pathogens15070753 (PMC13415161; doi:10.3390/pathogens15070753)

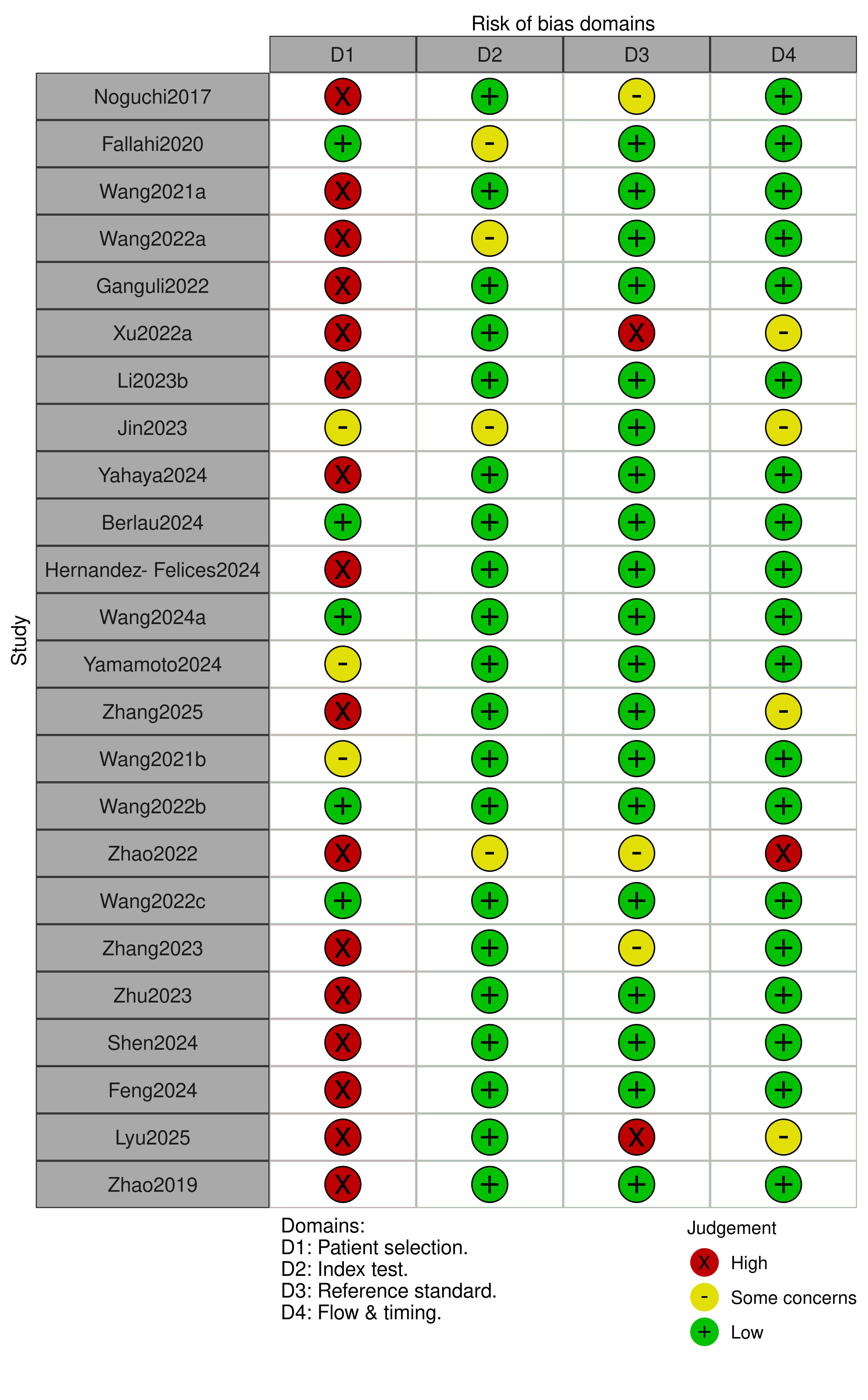

Supplement: Supplementary file 1 [file pathogens-15-00753-s001.zip › S3-Traffic light plot for risk of bias assessment in isothermal methods.jpeg]

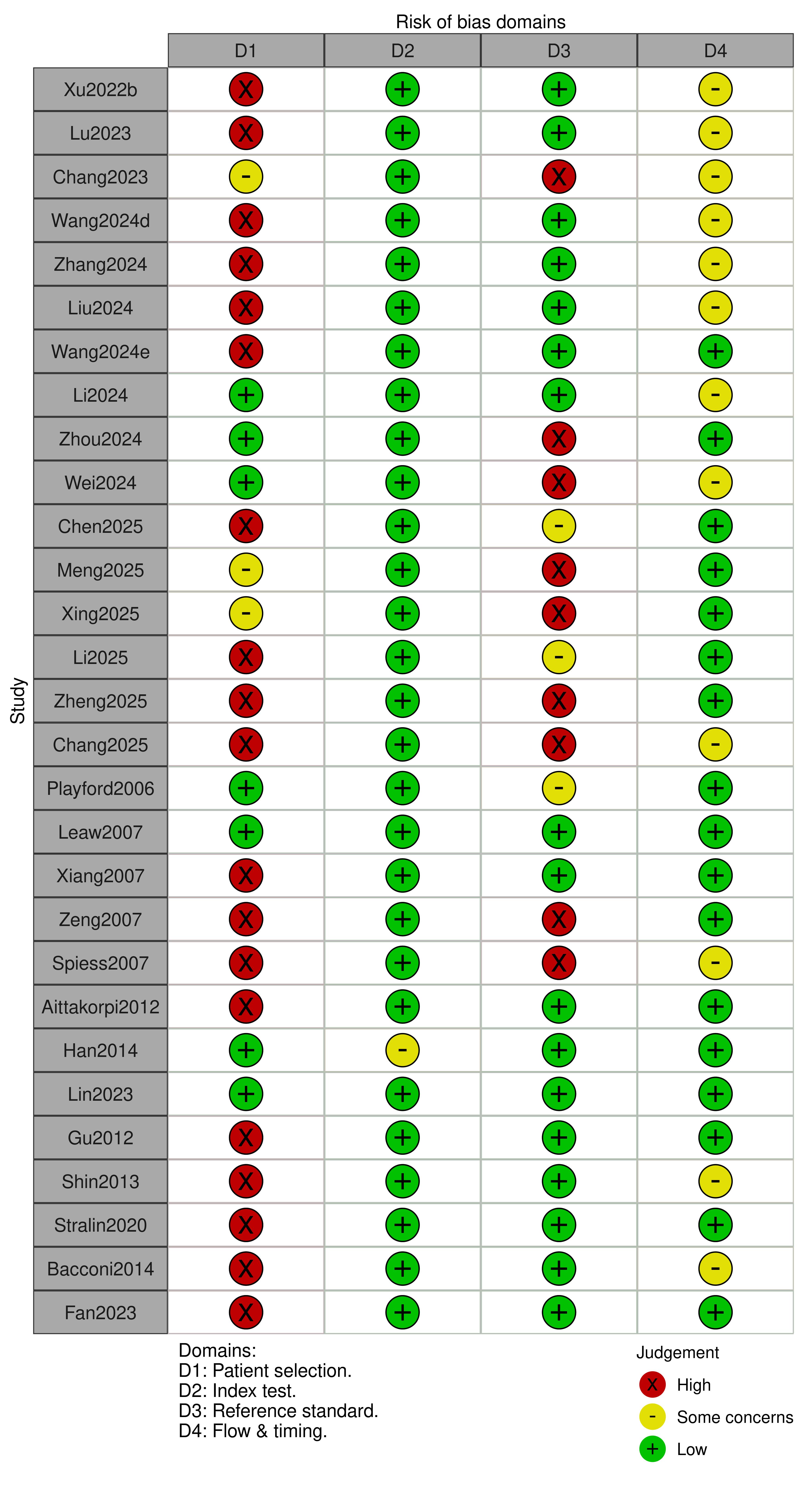

Supplement: Supplementary file 1 [file pathogens-15-00753-s001.zip › S4-Traffic light plot for risk of bias assessment in high throughput methods.jpeg]
